# A novel multiplex biomarker panel for profiling human acute and chronic kidney disease

**DOI:** 10.1038/s41598-023-47418-9

**Published:** 2023-12-01

**Authors:** Logan R. Van Nynatten, Michael R. Miller, Maitray A. Patel, Mark Daley, Guido Filler, Sigrun Badrnya, Markus Miholits, Brian Webb, Christopher W. McIntyre, Douglas D. Fraser

**Affiliations:** 1 Medicine, Western University, London, ON Canada; 2 Pediatrics, Western University, London, ON Canada; 3 Epidemiology and Biostatistics, Western University, London, ON N6A 3K7 Canada; 4 Computer Science, Western University, London, ON N6A 3K7 Canada; 5 The Vector Institute for Artificial Intelligence, Toronto, ON M5G 1M1 Canada; 6 Lawson Health Research Institute, London, ON Canada; 7 Thermo Fisher Scientific, Vienna, Austria; 8 Thermo Fisher Scientific, Rockford, IL USA; 9 Clinical Neurological Sciences, Western University, London, ON Canada; 10 Physiology and Pharmacology, Western University, London, ON Canada; 11 London Health Sciences Centre, Room C2-C82, 800 Commissioners Road East, London, ON N6A 5W9 Canada

**Keywords:** Kidney, Biomarkers

## Abstract

Acute and chronic kidney disease continues to confer significant morbidity and mortality in the clinical setting. Despite high prevalence of these conditions, few validated biomarkers exist to predict kidney dysfunction. In this study, we utilized a novel kidney multiplex panel to measure 21 proteins in plasma and urine to characterize the spectrum of biomarker profiles in kidney disease. Blood and urine samples were obtained from age-/sex-matched healthy control subjects (HC), critically-ill COVID-19 patients with acute kidney injury (AKI), and patients with chronic or end-stage kidney disease (CKD/ESKD). Biomarkers were measured with a kidney multiplex panel, and results analyzed with conventional statistics and machine learning. Correlations were examined between biomarkers and patient clinical and laboratory variables. Median AKI subject age was 65.5 (IQR 58.5–73.0) and median CKD/ESKD age was 65.0 (IQR 50.0–71.5). Of the CKD/ESKD patients, 76.1% were on hemodialysis, 14.3% of patients had kidney transplant, and 9.5% had CKD without kidney replacement therapy. In plasma, 19 proteins were significantly different in titer between the HC versus AKI versus CKD/ESKD groups, while NAG and RBP4 were unchanged. TIMP-1 (PPV 1.0, NPV 1.0), best distinguished AKI from HC, and TFF3 (PPV 0.99, NPV 0.89) best distinguished CKD/ESKD from HC. In urine, 18 proteins were significantly different between groups except Calbindin, Osteopontin and TIMP-1. Osteoactivin (PPV 0.95, NPV 0.95) best distinguished AKI from HC, and β2-microglobulin (PPV 0.96, NPV 0.78) best distinguished CKD/ESKD from HC. A variety of correlations were noted between patient variables and either plasma or urine biomarkers. Using a novel kidney multiplex biomarker panel, together with conventional statistics and machine learning, we identified unique biomarker profiles in the plasma and urine of patients with AKI and CKD/ESKD. We demonstrated correlations between biomarker profiles and patient clinical variables. Our exploratory study provides biomarker data for future hypothesis driven research on kidney disease.

## Introduction

Kidney injury continues to confer significant morbidity and mortality in the clinical setting, both in acute kidney injury (AKI) and chronic/end-stage kidney disease (CKD/ESKD). The incidence of AKI in hospitalized subjects exceeds 20%^1^. Elderly age and underlying kidney dysfunction predispose individuals to AKI, with circulatory disease and infection being the most common precipitant factors^1^. Moreover, AKI is independently associated with in-hospital mortality, length of stay, and healthcare cost^1,2^. Similarly, CKD has an enormous global burden with a prevalence of ~ 10–15%, resulting in 1.2 million deaths annually^3^. A substantial proportion of patients with CKD progress to ESKD, which confers a 13-fold increase in the rate of mortality, especially from cardiovascular disease^4,5^. Despite life-saving kidney replacement therapy (continuous kidney replacement therapy [CKRT], hemodialysis [HD] or peritoneal dialysis [PD]), most patients experience drastically impaired quality of life^6^, and only a small fraction of patients undergo kidney transplantation^7,8^. Moreover, critically-ill patients with kidney failure disproportionately experience negative outcomes. Intensive care unit (ICU) mortality is reported to be 23% in those with AKI, and 11% in those with ESKD^9^.

Despite such striking morbidity and mortality of kidney disease, there are few biomarkers to aid diagnostics and prognostics, especially in critically-ill patients^10^. Kidney biomarkers could aid disease surveillance, as well as act as triggers for therapeutic interventions^11,12^. Hence, the need to identify early biomarkers of kidney impairment, particularly those associated with clinically relevant parameters, is important. The number of biomarkers (serum and urine) associated with kidney injury has steadily increased with several proteins showing strong associations with kidney outcomes (e.g., KIM-1. NGAL)^13,14^. Yet, in isolation, each of these targets provides imperfect information to predict outcomes in acute or chronic kidney disease, especially early in disease. Given the inherent complexity of kidney physiology, it is unlikely that any single biomarker in isolation will have clinical utility for a broad scope of patients. For example, the dynamics of solute and electrolyte transport throughout the nephron will be highly variable between individuals, as well as the etiology of kidney injury (whether it be a glomerular, tubular, interstitial or vascular insult). Identifying a panel or series of biomarkers, from both plasma and urine that correlate with patient clinical and biochemical parameters may have enormous power to serve as biomarkers for a general population.

In this study, we employed a novel kidney multiplex panel consisting of 21 biomarkers in both plasma and urine of healthy control subjects, patients with AKI, and patients with CKD/ESKD. Our aims were: (1) to validate the novel kidney multiplex panel to detect biomarkers in plasma and urine, (2) to identify biomarkers that distinguish patients with AKI versus CKD/ESKD, (3) to identify rank orders and accuracies of the biomarkers to identify kidney disease, and (4) to assess correlations between these biomarkers with patient clinical and laboratory parameters.

## Methods

This study was approved by the Western University Human Research Ethics Board. This study was performed in accordance with ethical standards of the responsible committee on human experimentation with the Helsinki Declaration of 1975. Informed written consent was obtained from every participant.

Age- and sex-matched healthy control subjects, AKI patients (admitted to ICU at London Health Sciences Center, a tertiary university hospital in London, Ontario, Canada, in the context of COVID-19 infection) and CKD/ESKD patients were enrolled. Kidney disease was classified based on KDIGO practice guidelines^15,16^; AKI was defined as an increase in serum creatinine to 1.5 times baseline in the last 7 days; CKD was defined in either alteration in kidney structure or function for > 3 months (albuminuria > 3 mg/mmol, urine sediment abnormalities, electrolyte disturbance secondary to tubular disorders, structural abnormalities detected by imaging, kidney transplant history, eGFR < 60 mL/min/1.73 m^2^); and ESKD was defined as being on kidney replacement therapy. All AKI patients in the ICU who required kidney replacement therapy were initiated on continuous kidney replacement therapy (CKRT) as the modality of choice, while all patients with ESKD were on intermittent hemodialysis.

Patient characteristics included age, sex, BMI, comorbidities (hypertension, diabetes, coronary artery disease, congestive heart failure, cancer, chronic obstructive pulmonary disease, asthma, cirrhosis, chronic kidney disease, end stage kidney disease, hemodialysis, previous kidney transplant), hematology (hemoglobin, leukocytes, lymphocytes, thrombocytes), serum electrolytes (sodium, potassium, chloride, bicarbonate, calcium, phosphate, magnesium,) biochemistry (Prothrombin Time Test (PTT) and international normalized ratio (INR), glucose, bilirubin, Alanine transaminase (ALT), Aspartate transaminase (AST), Alkaline phosphatase (ALP), Gamma-glutamyl transpeptidase (GGT), lactate, C-reactive protein (CRP), ferritin, troponin I, lactate, D-Dimer, albumin, intact parathyroid hormone (iPTH)), and kidney function (serum urea, serum creatinine, serum cystatin C, urine sodium, urine creatinine, urine microalbumin/creatinine, and CKD-EPI estimated glomerular filtration rate [CKD-EPI eGFR]^17^). Need for dialysis as well as outcome (dead or alive) was recorded. Not all clinical variables were similarly available for all groups. No adjustments were made for missing values.

### Blood draws

Standard phlebotomy procedures were used to collect blood. Samples were obtained from central venous catheters in critically-ill ICU patients, via peripheral phlebotomy for healthy subjects and CKD patients, and via indwelling hemodialysis catheters for those patients on hemodialysis. Samples were immediately placed on ice and transferred to a negative pressure hood. After centrifugation, isolated plasma was divided in 250 µL aliquots and frozen at − 80 °C. All samples remained frozen until use to avoid freeze–thaw cycles. Maximum phlebotomy volumes were not exceeded. Blood was drawn from COVID-19 patients upon admission to ICU, and in ESKD patients, blood was drawn prior to initiation of routine hemodialysis. Fasting was not requested, nor initiated prior to blood draws.

### Urine collection

Urine was collected into sterile, screw-top containers. AKI patients had urine collected from indwelling urinary catheters before the initiation of CKRT (all patients produced urine). CKD/ESKD patients were asked to provide a midstream urine sample. In both scenarios, a portion of the collected urine was sent immediately to the hospital laboratory for processing, and the remaining urine was aliquoted, frozen at − 80 °C and stored in a secured biorepository until use.

### Kidney multiplex measurements

Concentrations of 21 kidney toxicity biomarkers were determined in human plasma and urine using two multiplexed immunoassay kits according to manufacturer’s instructions (Thermo Fisher Scientific): Human ProcartaPlex™ Kidney Toxicity Panel 1 (KIM-1, Calbindin, Renin, Osteoactivin, Clusterin (APO-J), GSTA1, NAG, RBP4, IL-18, IP-10, EGF, MCP-1 and VEGF-A) and Human ProcartaPlex™ Kidney Toxicity Panel 2 (Uromodulin, TFF-3, Cystatin C, NGAL, Osteopontin, α1-microglobulin, TIMP-1 and β2-microglobulin). Briefly, 50 µL of the Capture Bead Mix was added to each well of a 96-well flat bottom plate. After washing of capture beads, 25 µL of Universal Assay Buffer (1x) was added to each well, followed by 25 µL of undiluted plasma or urine sample (Human ProcartaPlex™ Kidney Toxicity Panel 1), 1:100 pre-dilution in Universal Assay Buffer (1x) (Human ProcartaPlex™ Kidney Toxicity Panel 2), prepared standards or blank. The plate was incubated at room temperature in the dark on a plate shaker at 600 rpm for 60 min. After incubation, the plate was washed and incubated with the Detection Antibody Mix (25 µL/well) for 30 min. Following a wash step to remove unbound antibody, 50 µL of Streptavidin-PE (SAPE) solution was added to each well and incubated for 30 min as above. After washing and addition of Reading Buffer, plates were processed on a compatible Luminex^®^ system. Both multiplexed immunoassay kits utilize Luminex^®^ xMAP™ fluorescent bead-based technology (Luminex Corp., 12212 Technology Blvd, Austin, TX, 78727, USA) and were quantified on a Bio-Plex™ 200 system (Bio-Rad Laboratories, 1000 Alfred Nobel Drive, Hercules, CA, 94547, USA).

### Conventional statistics

Medians (interquartile ranges [IQRs]) and frequency (%) were used to report baseline characteristics of healthy control subjects, AKI patients, and CKD/ESKD patients, for continuous and categorical variables, respectively (GraphPad Prism Version 8.4.0; San Diego, California, USA). As data were not normally distributed on Shapiro Wilk test, the non-parametric Mann–Whitney U test was used to compare two groups, whereas a Kruskal–Wallis test was used to compare three groups. Statistical significance was set at *P* < 0.05 following Bonferroni correction for multiple comparisons. Heat maps depicting Pearson correlation values between plasma and urine proteins with patient clinical variables were created in R (http://www.r-project.org) using the ggplot2 version 3.3.3 package. Significant correlations had R-values of ≥ 0.7 or ≤ − 0.7 for AKI and ≥ 0.4 or ≤ − 0.4 for CKD/ESKD, with a *P*-value of significance set at < 0.05. Not all data were fully available for all subgroups, as different clinical data were recorded.

### Machine learning

A Random Forest classifier, based on decision trees, was used for each cohort comparison and determined the ability to classify the participants into their respective groups based on the biomarker values. The Random Forest was built using Scikit-Learn (Python v3.10.4, Scikit-learn v1.1.1^18^, function = sklearn.ensemble.RandomForestClassifier). To reduce overfitting and maintain a conservative model, three-fold cross-validation with a Random Forest of 10 trees and a maximum depth of three was used^19^. The biomarkers were then investigated to determine their importance in differentiating renal disease. As this was not to determine the predictive ability but to interrogate the biomarkers from a physiological perspective, a single Random Forest model with 1000 trees and no specified maximum depth was constructed. The Random Forest model was fit on the complete dataset to ensure the model incorporated the impact of all features for all samples. The biomarkers’ order of importance was determined by the inherent ability of Scikit-Learn Random Forest model, which uses Gini Importance.

Receiver operating characteristic (ROC) curves using logistic regression were conducted to determine the sensitivity and specificity of individual proteins in their respective comparison (Python v3.10.4, Scikit-learn v1.1.1^20^, function = sklearn.linear_model.LogisticRegression). Area-under-the-curve (AUC) was calculated as an aggregate measure of biomarker performance across all possible classification thresholds^19^. The F1 score was determined as the harmonic mean of precision and recall. A high F1 score indicated that both precision and recall were high. A bootstrap method of 1000 repetitions with resampling with replacement and three-fold cross-validation was used to determine the average F1 score, positive predictive value (PPV), and negative predictive value (NPV), as well as to determine the average ROC curve AUC and build a 95% confidence interval. The biomarker data were visualized with a nonlinear dimensionality reduction on the full, reduced, and optimal datasets using the t-distributed stochastic nearest neighbor embedding (t-SNE) algorithm (Python v3.10.4, Scikit-learn v1.1.1^20^, function = sklearn.manifold.TSNE). t-SNE assumes that the ‘optimal’ representation of the data lies on a manifold with complex geometry, but a low dimension, embedded in the full-dimensional space of the raw data^21^.

### Ethics approval and consent to participate

This study was approved by the Western University, Human Research Ethics Board (HREB): kidney patients (HREB #6970, renewed March 17, 2021) and volunteer healthy control subjects (HREB #16986E, renewed March 9, 2021).

## Results

Demographic, clinical and laboratory parameters for AKI (in the context of COVID-19 infection admitted to the ICU) and CKD/ESKD patients are depicted in Table 1. Of AKI patients, median (IQR) for MODS was 7.0 (6.0–7.0), SOFA was 8.0 (8.0–10.5) and APACHEII was 15.0 (13.0–20.0). Median (IQR) for the AKIN score was 3.0 (1.5–3.0), suggesting significant AKI in critically-ill patients with COVID-19. The mean arterial pressure median (IQR) upon admission to ICU was 79 (67–100) mmHg, but was recorded on vasopressors. Of the CKD/ESKD patients, 16 were on intermittent hemodialysis, 3 patients had kidney transplants, and 2 patients had CKD without receiving kidney replacement therapy. The CKD-EPI median (IQR) GFR was 52 (14–64) ml/min indicating Stage III CKD. All healthy control subjects and CKD/ESKD patients survived the study period, whereas the mortality of AKI patients in the ICU was 87.5%.

**Table 1:** Patient demographic, clinical and laboratory data.

Clinical variables	Healthy control	AKI (COVID-19)	CKD/ESKD
n	8	8	21
Age, yr, median (IQR)	51.0 (38.5–57.5)	65.5 (58.5–73.0)	65.0 (50.0–71.5)
Sex, female: male	5:3	1:7	7:14
BMI, median (IQR)	26.4 (21.6–30.5)	29.1 (27.4–33.3)	28.3 (23.9–33.5)
MODS, median (IQR)	–	7.0 (6.0–7.0)	–
SOFA, median (IQR)	–	8.0 (8.0–10.5)	–
APACHEII, median (IQR)	–	15.0 (13.0–20.0)	–
AKIN, median (IQR)	–	3.0 (1.5–3.0)	–
Comorbidities, n (%)			
Hypertension	–	4 (50.0)	18 (85.7)
Diabetes	–	4 (50.0)	8 (38.1)
CAD	–	1 (12.5)	7 (33.3)
CHF	–	0 (0)	5 (23.8)
Cancer	–	0 (0)	1 (4.7)
COPD	–	0 (0)	2 (9.5)
Asthma	–	0 (0)	0 (0)
Cirrhosis	–	0 (0)	1 (4.7)
CKD	–	0 (0)	2 (9.5)
ESRD	–	0 (0)	19 (90.5)
IHD	–	–	16 (76.1)
Transplant	–	–	3 (14.3)
Interventions, n (%)			
Intubation	–	8 (100)	–
Vasopressors	–	8 (100)	–
Antibiotics	–	8 (100)	–
Steroid	–	8 (100)	–
Tocilizumab	–	1 (12.5)	–
Haematology, median (IQR)			
Hemoglobin	139.0 (130.5–159.5)	86.0 (84.0–114.0)	116.0 (106.0–135.0)
Leukocytes	–	14.6 (11.6–20.5)	6.7 (5.5–9.2)
Lymphocytes	–	0.7 (0.6–1.8)	1.3 (0.8–2.0)
Platelets	–	237.0 (216.5–270.5)	235.0 (181.0–269.0)
Electrolytes, median (IQR)			
Sodium	140.5 (139.0–141.5)	135.0 (133.0–141.0)	139.0 (136.5–141.0)
Potassium	3.9 (3.6–4.2)	4.3 (3.9–4.6)	4.2 (3.8–4.9)
Chloride	–	96.0 (95.0–98.5)	93.5 (91.5–95.5)
Bicarbonate	25.0 (24.0–27.0)	24.5 (22.5–33.0)	26.0 (25.0–28.5)
Calcium	2.3 (2.3–2.5)	2.0 (1.9–2.1)	2.4 (2.3–2.4)
Phosphate	0.9 (0.8–1.2)	1.2 (1.0–1.7)	1.5 (1.2–1.8)
Magnesium	–	0.9 (0.8–1.1)	–
Parathyroid hormone	4.0 (3.5–4.5)	–	25.6 (15.0–64.5)
Biochemistry, median (IQR)			
INR	–	1.2 (1.1–1.3)	–
PTT	–	28.5 (25.0–67.0)	–
Glucose	–	7.0 (5.8–9.2)	5.8 (5.2–9.0)
Bilirubin	–	8.8 (6.0–13.9)	–
AST	–	48.0 (40.0–79.5)	–
ALT	–	36.5 (30.0–102.0)	–
ALP	–	131.5 (106.5–147.5)	–
GGT	–	139.0 (73.0–510.0)	–
LDH	–	456.5 (351.0–648.0)	–
CRP	–	131.5 (40.3–180.5)	3.8 (1.7–14.6)
Ferritin	–	1106.5 (796.5–1936.0)	–
Troponin	–	61.0 (20.5–191.0)	–
Lactate	–	1.5 (1.2–1.9)	–
D-Dimer	–	3222.5 (2578.5–7029.5)	–
Albumin	–	24.5 (21.5–25.5)	–
Renal function, median (IQR)			
Serum Urea	4.9 (3.9–6.3)	16.5 (12.3–24.0)	13.6 (8.4–17.1)
Serum Creatinine	68.5 (58.5–75.5)	85.0 (70.0–241.5)	455.0 (268.5–557.0)
Serum Cystatin-C	0.8 (0.8–0.9)	–	5.1 (3.7–5.6)
Urine Creatinine	17.5 (10.8–23.9)	4.1 (2.7–8.1)	7.9 (4.1–9.8)
Urine Sodium	86.5 (72.0–116.5)	81.5 (29.0–109.0)	68.0 (41.5–84.0)
Urine Microalbumin/creatinine	0.4 (0.4–1.0)	56.5 (43.0–111.0)	50.5 (11.7–308.0)
CKD-EPI Creatinine	102.5 (86.0–107.0)	–	12.0 (8.5–17.0)
CKD-EPI Creatinine/cystatin	100.0 (93.0–104.0)	–	10.0 (7.0–12.5)
CKD-EPI Cystatin-C	89.5 (94.5–98.5)	–	8.0 (7.0–11.5)
LOS, median (IQR)			
ICU	–	19.5 (13.0–27.5)	–
Hospital	–	19.5 (13.0–28.5)	–
Outcome, n (%)			
Alive	8 (100)	1 (12.5)	21 (100)
Dead	0 (0)	7 (87.5)	0 (0)

*MODS* multiple organ dysfunction score, *SOFA* sequential organ failure assessment, *APACHE* acute physiology and chronic health evaluation, *AKIN* acute kidney injury score, *LOS* length of stay.

Our study utilized a novel kidney multiplex panel for 21 biomarker proteins (Supplementary Table 1). When comparing plasma levels of biomarkers in healthy control subjects, AKI, and CKD/ESKD, all protein concentrations were significantly different between the three groups, except N = Acetyl-Beta-d-Glycosaminidase (NAG) and Retinol-binding protein 4 (RBP4) (Table 2). When comparing urine biomarker levels, all proteins were significantly different in titer between healthy control subjects, AKI and CKD/ESKD, except Calbindin, Osteopontin and TIMP metallopeptidase inhibitor 1 (TIMP-1) (Table 3). When comparing individual groups in two-way analyses, statistical differences were again noted (Supplementary Tables 2 and 3). Three patients had plasma levels of biomarkers compared pre- and post-dialysis on Week 1 and Week 14 of study, with no significant differences in titer pre- and post-dialysis (Supplementary Tables 4 and 5). The PPV and NPV for each protein as biomarkers for renal disease are reported in Supplementary Tables 6–9.

**Table 2:** Plasma biomarker values (pg/ml).

Protein	Healthy controls (n = 8)	AKI (COVID-19) (n = 8)	CKD/ESKD (n = 21)	*P*-value
KIM-1	274.5 (230.6–288.4)	742.9 (625.6–1324.1)	582.5 (278.5–789.4)	**0.002**
NAG	0 (0–0)	0 (0–1218.2)	0 (0–0)	0.299
Calbindin	3.6 (0–20.0)	88.9 (59.2–275.9)	37.5 (12.5–70.7)	**< 0.001**
GSTA1	202.8 (157.4–314.9)	652.0 (505.0–718.6)	436.5 (201.8–540.7)	**< 0.001**
Osteoactivin	195.3 (155.1–217.0)	501.5 (380.6–1217.9)	493.0 (324.3–883.5)	**0.003**
Renin	37.1 (27.1–59.6)	670.4 (342.9–2539.6)	137.3 (99.5–326.7)	**< 0.001**
Clusterin	264,584 (221,595–351,445)	593,503 (448,604–709,786)	470,516 (328,132–677,194)	**0.004**
RBP4	77,963 (54,925–125,445)	221,824 (143,659–404,136)	203,704 (75,150–342,768)	0.083
IL-18	24.5 (19.3–29.9)	185.3 (162.3–249.4)	84.6 (39.7–109.5)	**< 0.001**
IP-10	4.4 (3.7–5.5)	95.5 (71.8–146.2)	12.3 (7.1–16.8)	**< 0.001**
EGF	2.1 (0.1–5.3)	19.3 (15.3–23.3)	13.3 (1.8–17.8)	**0.001**
MCP-1	12.0 (8.6–15.0)	227.8 (57.7–424.3)	33.3 (20.0–66.2)	**< 0.001**
VEGF-A	35.6 (34.0–41.6)	430.4 (217.2–678.5)	673.4 (286.2–806.1)	**< 0.001**
Uromodulin	103.0 (0–199.7)	605.6 (0–776.0)	1308.5 (674.7–1617.3)	**< 0.001**
α1-microglobulin	775.3 (520.3–1122.8)	479.7 (294.0–914.4)	4318.6 (2782.1–5212.4)	**< 0.001**
TFF3	3.1 (2.1–4.6)	13.8 (7.9–33.1)	55.1 (24.2–62.4)	**< 0.001**
Osteopontin	17.8 (15.3–23.4)	308.3 (243.3–527.8)	55.9 (29.5–78.1)	**< 0.001**
Cystatin C	207.9 (173.8–230.8)	269.86 (246.2–532.8)	563.4 (409.4–700.8)	**< 0.001**
NGAL	73.4 (58.4–110.7)	202.6 (161.9–355.0)	359.4 (259.6–520.4)	**< 0.001**
β2-microglobulin	913.0 (751.0–1136.7)	3700.7 (2361.7–4849.4)	4972.3 (3114.4–5763.0)	**< 0.001**
TIMP-1	34.5 (32.4–41.5)	172.6 (130.1–278.3)	88.1 (62.9–98.3)	**< 0.001**

Data are indicated by median (IQR).

Bold indicates statistically significant P values.

**Table 3:** Urine biomarker values (pg/ml).

Protein	Healthy control (n = 8)	AKI (COVID-19) (n = 8)	CKD/ESKD (n = 18)	*P*-value
KIM-1	305.2 (276.4–1011.3)	1286.6 (466.7–3600.0)	1235.7 (652.0–3107.1)	**0.047**
NAG	0 (0–0)	14.0 (0.7–46.8)	1.4 (0–70.7)	**0.025**
Calbindin	2368.6 (1413.4–5141.2)	3688.5 (1106.4–6309.2)	1450.7 (678.0–3641.2)	0.271
GSTA1	6.6 (2.5–17.5)	132.1 (111.9–271.8)	189.5 (18.7–246.9)	**< 0.001**
Osteoactivin	36.1 (15.7–43.3)	86.4 (60.3–320.1)	179.9 (81.7–363.1)	**0.003**
Renin	8.8 (1.9–21.1)	605.3 (131.6–2431.6)	65.0 (18.5–319.6)	**< 0.001**
Clusterin	39,599 (26,401–73,940)	177,660 (24,062–506,175)	260,515 (113,239–552,746)	**0.027**
RBP4	2253 (939–3212)	66,742 (5055–131,315)	718,316 (14,892–718,316)	**< 0.001**
IL-18	19.0 (10.9–29.4)	83.9 (30.0–174.7)	64.5 (33.8–95.8)	**0.024**
IP-10	3.0 (2.0–8.9)	55.5 (25.0–144.3)	15.4 (5.4–32.8)	**0.006**
EGF	6538 (4842–7408)	3951 (2956–5779)	1829 (1394–2743)	**< 0.001**
MCP-1	152.7 (75.7–184.2)	625.1 (292.8–1538.9)	771.2 (448.9–1044.0)	**0.001**
VEGF-A	477.7 (149.4–689.5)	1295.4 (330.6–3004.0)	2834.9 (787.5–6492.9)	**0.010**
Uromodulin	44,794 (26,420–161,855)	31,602 (7414–43,024)	3485 (1792–8430)	**0.001**
α1-microglobulin	298.5 (202.4–350.7)	162.5 (104.0–538.2)	2482.7 (736.4–2743.4)	**< 0.001**
TFF3	45.3 (33.3–62.0)	274.8 (84.7–304.5)	228.6 (150.9–442.5)	**0.003**
Osteopontin	1127 (625–1685)	1089 (293–3191)	488 (259–822)	0.107
Cystatin C	37.4 (20.7–62.2)	143.2 (27.3–2283.8)	1335.3 (96.5–1667.1)	**0.013**
NGAL	30.3 (14.5–54.0)	97.8 (37.0–477.4)	456.0 (263.6–570.3)	**0.003**
β2-microglobulin	84.5 (42.5–189.3)	3391.0 (516.5–5687.2)	13,412.0 (670.4–19,924.8)	**< 0.001**
TIMP-1	10.6 (6.5–12.9)	12.9 (3.4–28.4)	19.9 (9.5–31.0)	0.255

Data are indicated by median (IQR).

Bold indicates statistically significant P values.

ROC curves were generated to identify the ability of these 21 biomarkers to differentiate either patients with AKI or CKD/ESKD from healthy control subjects (Tables 4 and 5). F1 statistics were calculated to verify biomarker precision and accuracy in differentiating kidney disease patients from healthy control subjects. In plasma, KIM-1, Calbindin, glutathione S-transferase alpha 1 (GSTA1), Renin, Clusterin, Interleukin 18 (IL-18), IP-10, Epidermal growth factor (EGF), MCP-1, VEGF-A, TFF3, Osteopontin, NGAL, β2-microglobulin and TIMP-1 were statistically able to differentiate AKI from healthy control subjects (Table 4). Also, plasma levels of Osteoactivin, Renin, IL-18, IP-10, MCP-1, VEGF-A, Uromodulin, α1-microglobulin, TFF3, Osteopontin, Cystatin C, NGAL, β2-microglobulin, and TIMP-1 were statistically able to differentiate CKD/ESKD from healthy control subjects (Table 4).

**Table 4:** Plasma biomarker logistic regression ROC curve, F1, and *P*-value.

Protein	AKI (COVID-19) versus control			CKD/ESKD versus control		
	ROC curve AUC (95%CI)	F1	*P*-value	ROC curve AUC (95%CI)	F1	*P*-value
KIM-1	1.00 (1.00–1.00)	1.00	**0.003**	0.80 (0.80–0.81)	0.80	0.282
NAG	0.60 (0.59–0.61)	0.32	1.000	0.51 (0.51–0.51)	0.84	1.000
Calbindin	0.99 (0.98–0.99)	0.90	**0.026**	0.77 (0.76–0.77)	0.81	0.577
GSTA1	1.00 (1.00–1.00)	0.96	**0.003**	0.76 (0.76–0.77)	0.80	0.666
Osteoactivin	0.91 (0.91–0.92)	0.75	0.062	0.86 (0.85–0.87)	0.89	**0.026**
Renin	1.00 (1.00–1.00)	0.99	**0.003**	0.91 (0.91–0.91)	0.88	**0.006**
Clusterin	0.94 (0.93–0.94)	0.66	**0.039**	0.83 (0.83–0.84)	0.84	0.104
RBP4	0.50 (0.48–0.52)	0.32	0.434	0.50 (0.49–0.52)	0.40	1.000
IL-18	1.00 (1.00–1.00)	0.96	**0.003**	0.92 (0.91–0.92)	0.89	**0.015**
IP-10	1.00 (1.00–1.00)	0.96	**0.003**	0.85 (0.84–0.86)	0.88	**0.048**
EGF	1.00 (1.00–1.00)	0.99	**0.019**	0.77 (0.77–0.78)	0.80	0.586
MCP-1	1.00 (1.00–1.00)	0.92	**0.003**	0.96 (0.96–0.96)	0.93	**< 0.001**
VEGF-A	1.00 (1.00–1.00)	0.99	**0.003**	0.94 (0.94–0.95)	0.93	**0.001**
Uromodulin	0.73 (0.72–0.74)	0.64	1.000	0.97 (0.97–0.97)	0.92	**0.003**
α1-microglobulin	0.64 (0.62–0.65)	0.53	1.000	0.96 (0.96–0.97)	0.92	**< 0.001**
TFF3	0.98 (0.98–0.99)	0.89	**0.007**	0.97 (0.96–0.97)	0.96	**0.003**
Osteopontin	1.00 (1.00–1.00)	1.00	**0.003**	0.92 (0.91–0.92)	0.89	**0.005**
Cystatin C	0.90 (0.89–0.91)	0.76	0.098	0.97 (0.97–0.97)	0.94	**< 0.001**
NGAL	0.95 (0.95–0.96)	0.87	**0.023**	0.96 (0.96–0.96)	0.95	**< 0.001**
β2-microglobulin	1.00 (1.00–1.00)	0.98	**0.003**	0.98 (0.98–0.98)	0.96	**< 0.001**
TIMP-1	1.00 (1.00–1.00)	1.00	**0.003**	0.97 (0.97–0.97)	0.93	**< 0.001**

*p*-value—Bonferroni Corrected Mann–Whitney.

Bold indicates statistically significant P values.

**Table 5:** Urine biomarker logistic regression ROC curve, F1, and *P-value*.

Protein	AKI (COVID-19) versus control			CKD/ESKD versus control		
	ROC curve AUC (95%CI)	F1	*P*-value	ROC curve AUC (95%CI)	F1	*P*-value
KIM-1	0.74 (0.73–0.75)	0.58	1.000	0.81 (0.80–0.81)	0.82	0.279
NAG	0.88 (0.87–0.88)	0.78	0.096	0.73 (0.72–0.73)	0.78	0.679
Calbindin	0.52 (0.51–0.54)	0.44	1.000	0.58 (0.57–0.59)	0.79	1.000
GSTA1	1.00 (1.00–1.00)	0.95	**0.020**	0.92 (0.92–0.93)	0.85	**0.016**
Osteoactivin	1.00 (1.00–1.00)	0.93	**0.003**	0.86 (0.86–0.87)	0.83	0.056
Renin	0.94 (0.93–0.94)	0.86	**0.039**	0.88 (0.88–0.89)	0.83	**0.028**
Clusterin	0.71 (0.70–0.73)	0.64	1.000	0.83 (0.83–0.84)	0.82	0.132
RBP4	0.86 (0.85–0.87)	0.75	0.310	0.94 (0.93–0.94)	0.87	**0.009**
IL-18	0.79 (0.78–0.80)	0.65	0.796	0.79 (0.78–0.80)	0.83	0.194
IP-10	0.87 (0.86–0.88)	0.81	0.219	0.79 (0.78–0.80)	0.81	0.332
EGF	0.82 (0.81–0.83)	0.51	0.591	0.94 (0.93–0.94)	0.88	**0.003**
MCP-1	0.87 (0.86–0.88)	0.74	0.219	0.95 (0.95–0.95)	0.88	**0.001**
VEGF-A	0.59 (0.58–0.60)	0.49	1.000	0.85 (0.84–0.86)	0.82	0.070
Uromodulin	0.62 (0.61–0.64)	0.11	1.000	0.90 (0.89–0.91)	0.36	**0.005**
α1-microglobulin	0.49 (0.48–0.50)	0.44	1.000	0.94 (0.94–0.95)	0.89	**0.009**
TFF3	0.77 (0.76–0.78)	0.80	1.000	0.96 (0.95–0.96)	0.92	**0.001**
Osteopontin	0.49 (0.48–0.51)	0.43	1.000	0.72 (0.71–0.73)	0.82	1.000
Cystatin C	0.64 (0.63–0.65)	0.55	1.000	0.88 (0.88–0.88)	0.84	**0.028**
NGAL	0.76 (0.75–0.77)	0.66	1.000	0.90 (0.90–0.91)	0.88	**0.012**
β2-microglobulin	0.86 (0.85–0.86)	0.81	0.310	0.94 (0.93–0.94)	0.90	**0.003**
TIMP-1	0.49 (0.48–0.51)	0.44	1.000	0.64 (0.63–0.65)	0.78	1.000

*p*-value—Bonferroni Corrected Mann–Whitney.

Bold indicates statistically significant P values.

In urine, there were fewer biomarkers that differentiated AKI and CKD/ESKD from healthy control subjects (Table 5). Using ROC curve analyses, biomarkers that differentiated AKI from healthy control subjects included GSTA1, Osteoactivin, and Renin, whereas GSTA1, Renin, RBP4, EGF, MCP-1, Uromodulin, α1-microglobulin, TFF3, Cystatin C, NGAL and β2-microglobulin differentiated CKD/ESKD patients from healthy control subjects.

The 21 plasma and urine biomarkers were investigated using conservative Random Forest modelling to determine their classification ability in distinguishing the different renal conditions. Separate Random Forest models were created to pairwise classify healthy control subjects, AKI patients (COVID-19), and CKD/ESKD patients, as well as a three-class model with all patients (Supplementary Table 10). The plasma biomarkers performed well with high classification ability for all pairwise cohorts (balanced accuracy > 0.90, ROC AUC > 0.93, F1 Score > 0.92), as well as the three-class model (accuracy = 0.89, ROC AUC = 0.97). The urine biomarkers performed moderately well when used to compare healthy control subjects to CKD/ESKD patients (balanced accuracy = 0.83, ROC AUC = 0.94, F1 = 0.83); however, the other pairwise and three-class comparisons performed poorly.

**Figure 1:**
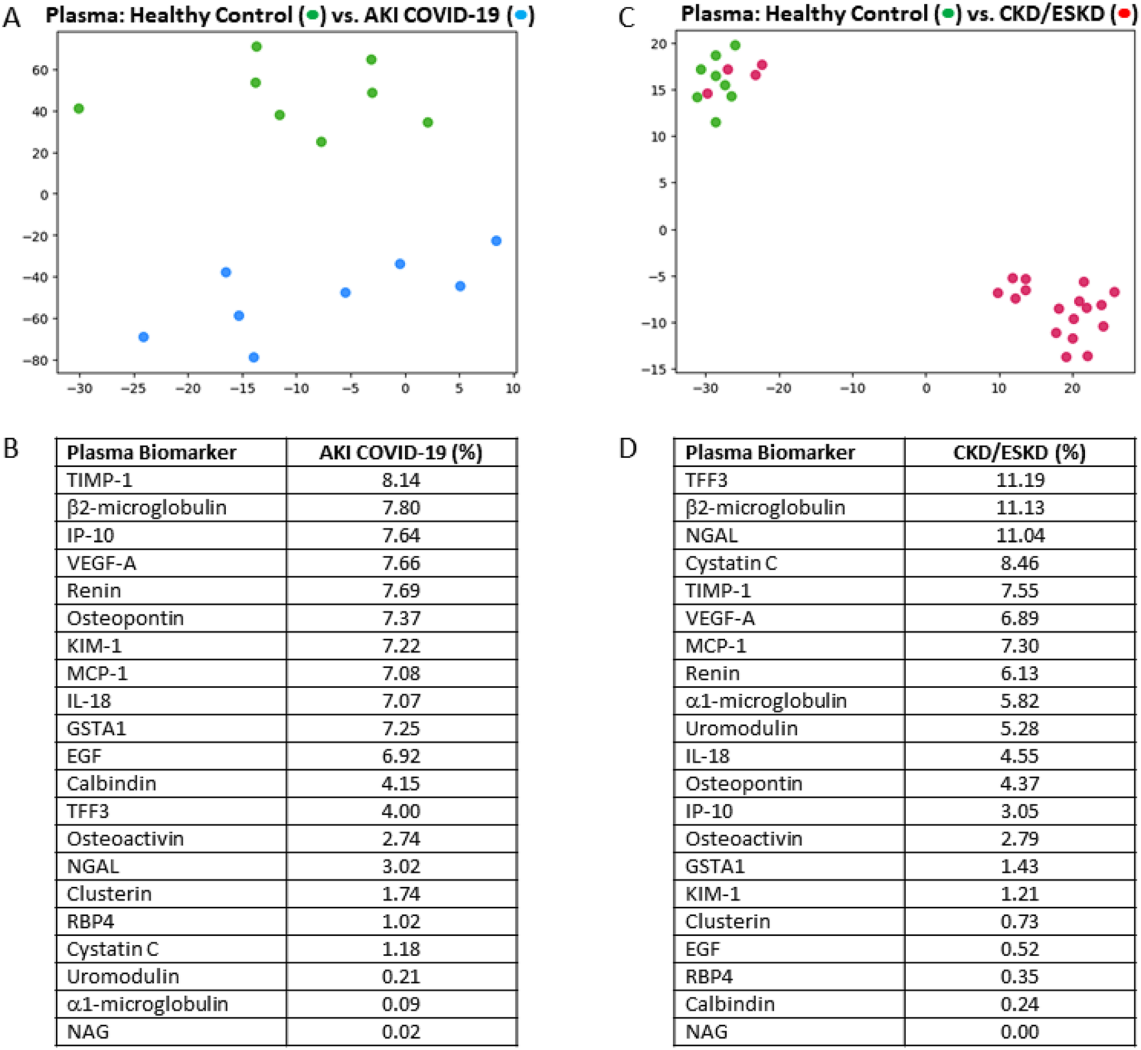
Plasma biomarkers accurately differentiate acute and chronic/end-stage kidney disease from healthy controls. In the upper section, t-SNE plots depict the separation between acute kidney injury (AKI) patients or chronic/end-stage (CKD/ESKD) kidney patients and healthy controls (HC). Subjects plotted in 2D following dimensionality reduction of their respective proteomes by t-SNE. Axes are dimensionless. The dimensionality reduction shows that based on plasma proteome, the two cohorts were distinct and easily separable.** A**) Blue dots represent AKI patients and green dots represent HC subjects.** B**) Plasma biomarkers distinguishing AKI patients from HC subjects in order of importance **C**) Pink dots represent CKD/ESKD and green dots represent HC subjects. Four CKD/ESKD patients visually resemble HC subjects (refer to Supplementary Table 6).** D**) Plasma biomarkers distinguishing CKD/ESRD patients from HC subjects in order of importance.

**Figure 2:**
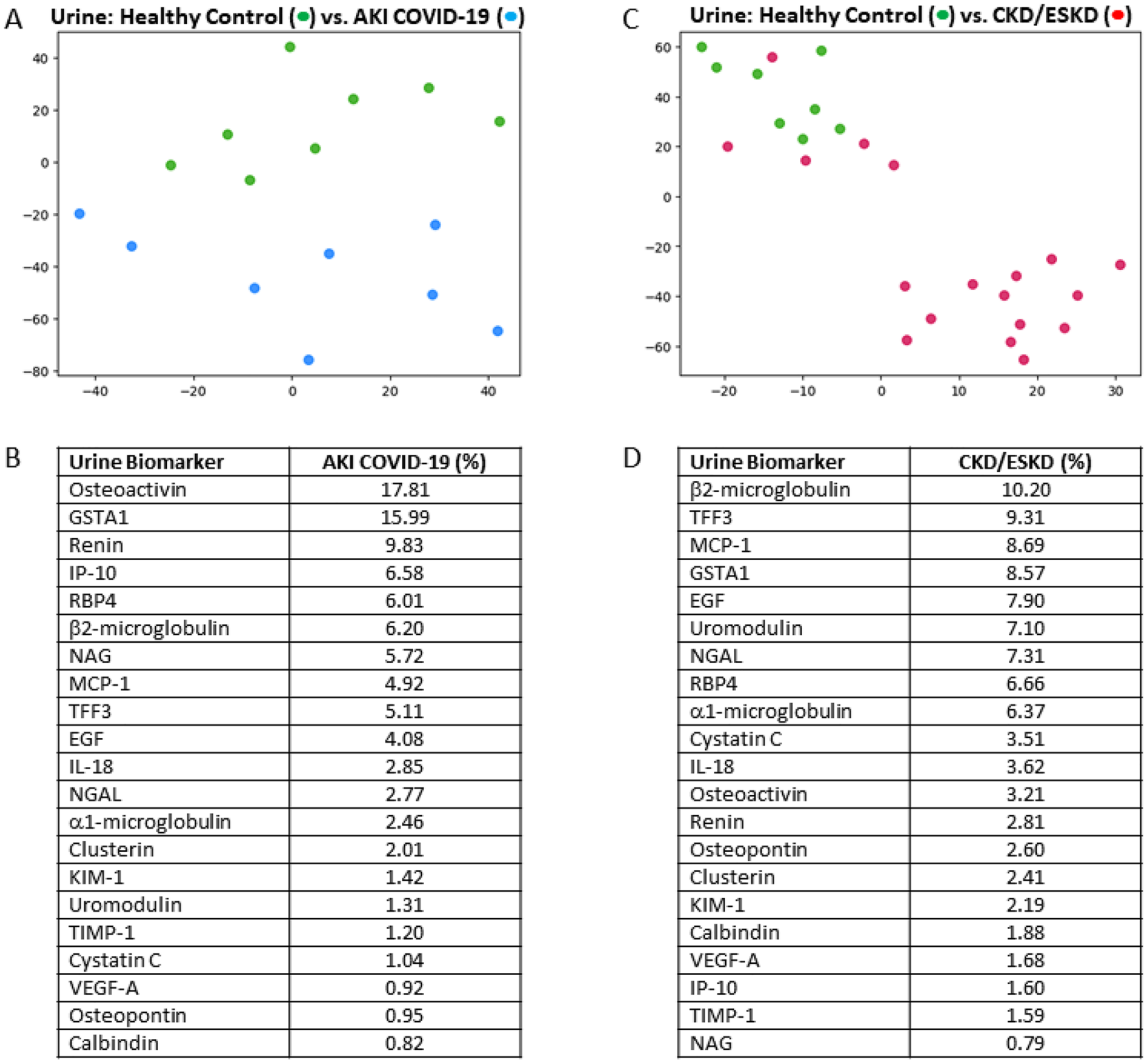
Urine biomarkers accurately differentiate acute and chronic/end-stage kidney disease from healthy controls. In the upper section, t-SNE plots depict the separation between acute kidney injury (AKI) patients or chronic/end-stage (CKD/ESKD) kidney patients and healthy controls (HC). Subjects plotted in 2D following dimensionality reduction of their respective proteomes by t-SNE. Axes are dimensionless. The dimensionality reduction shows that based on urine proteome, the two cohorts were distinct and easily separable.** A**) Blue dots represent AKI patients and green dots represent HC subjects.** B**) Urine biomarkers distinguishing AKI patients from HC subjects in order of importance.** C**) Pink dots represent CKD/ESKD patients and green dots represent healthy controls. Five CKD/ESKD patients visually resembled HC subjects (refer to Supplementary Table 6).** D**) Urine biomarkers distinguishing CKD/ESRD patients from HC subjects in order of importance.

Clustering analysis confirmed that the plasma and urine proteomes of healthy control subjects were distinct and easily separable from those patients with kidney disease as visualized on t-SNE plots (Figs. 1A,C and 2A,C). Interestingly, plasma obtained from four CKD/ESKD patients and their respective urine samples, shared biomarker signatures that visually resembled healthy control subjects (Figs. 1C and 2C); their plasma biomarker profiles were significantly different than the remaining 17 patients in the CKD/ESKD group (Supplementary Table 11). Specifically, Clusterin, VEGF-A, TIMP-1, Osteoactivin, β2-microglobulin, TFF3, Cystatin C, and α1-microglobulin all had significantly lower titers in the four CKD/ESKD patients who more closely resembled healthy control subjects. Of these four patients, none were on kidney replacement therapy and three were kidney transplant recipients (the remaining patient had CKD secondary to autosomal dominant polycystic kidney disease).

A rank order identified which proteins were most likely to differentiate kidney disease from healthy control subjects. In plasma, TIMP-1, β2-microglobulin, IP-10, VEGF-A, and Renin were the top five ranked proteins to distinguish AKI (Fig. 1A,B), whereas TFF3, β2-microglobulin, NGAL, Cystatin C and TIMP-1 were the top ranked proteins to distinguish CKD/ESKD (Fig. 1C,D). TIMP-1 and β2-microglobulin were common in their increased ability to differentiate either AKI or CKD/ESED from healthy control subjects.

In urine, Osteoactivin GSTA-1, Renin, IP-10, and RBP4 were the top 5 ranked proteins that differentiated healthy control subjects from AKI patients (Fig. 2A,B), whereas β2-microglobulin, TFF3, MCP-1, GSTA1 and EGF were the top proteins that differentiated healthy control subjects from those with CKD/ESKD (Fig. 2C,D). Only urine GSTA1 was common among both AKI and CKD/ESKD patients for differentiating kidney disease from healthy control subjects.

Among either urine or plasma, β2-microglobulin was the most shared biomarker that differentiated kidney disease from healthy control subjects (top 5 ranked proteins). NAG was consistently the lowest ranked protein in its ability to differentiate kidney disease from healthy control subjects. The plasma and urine biomarker profile of AKI as compared to CKD/ESKD patients was less distinct on t-SNE analysis, although still easily separable (Supplementary Fig. 1). Osteopontin, TIMP-1, IP-10, α1-microglobulin, and IL-18 were the top ranked biomarkers in plasma distinguishing AKI versus CKD/ESKD (Supplementary Fig. 1A,B). In urine, α1-microglobulin, Renin, IP-10, EGF and Uromodulin were the top 5 ranked proteins that distinguished AKI from CKD/ESKD subjects (Supplementary Fig. 1C,D).

**Figure 3:**
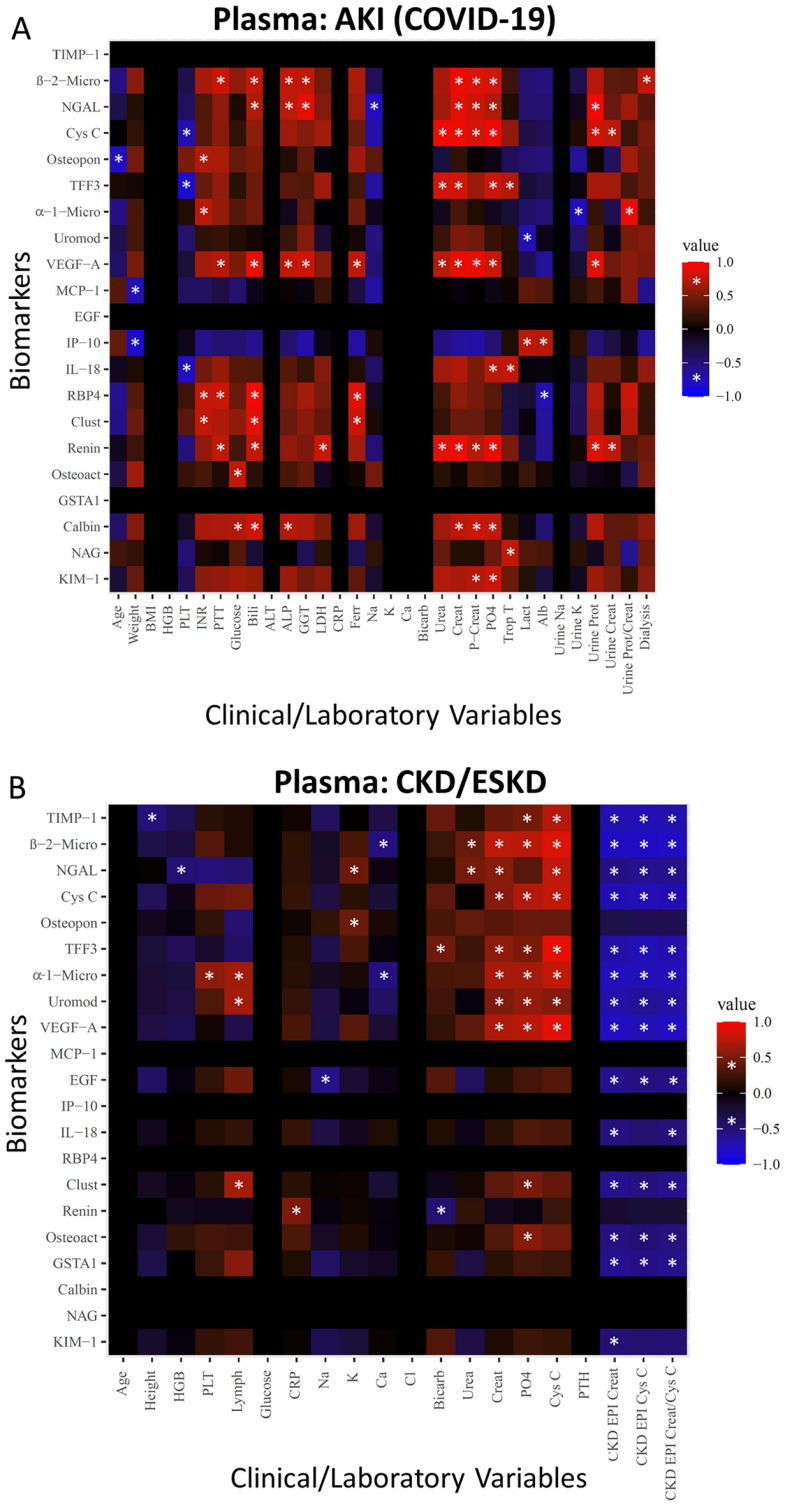
Correlations between plasma biomarker expression and acute and chronic/end-stage kidney disease patient parameters. Heat maps of rank-based classifying proteins reported in Fig. 1 in acute kidney injury (**A**) and chronic/end-stage kidney disease (**B**) is illustrated (y -axis) along with patient parameters (x-axis). Only proteins that showed a significant correlation (*P* ≤ 0.05) with at least one parameter are illustrated. Significant correlations had a Pearson R-value of ≥ 0.7 or ≤ -0.7 for AKI, ≥ 0.4 or ≤ -0.4 for CKD/ESKD, and *p*-value < 0.05, denoted by *. Positive correlations are depicted in red and negative correlations in blue. Abbreviations: BMI-body mass index; HGB-hemoglobin; PLT-platelet; Lymph-lymphocytes; INR-international normalized ratio; PTT-partial thromboplastin time; Bili-total bilirubin; ALT-alanine aminotransferase; ALP-alkaline phosphatase; GGT-gamma glutamyl transferase; LDH-lactate dehydrogenase; CRP-C reactive protein; Ferr-ferritin; Na-sodium; K-potassium; Ca-calcium; Bicarb-bicarbonate; Cl-chloride; Creat-creatinine; P-Creat-peak creatinine; PO4-phosphate; Trop-troponin; Lact-lactate; Alb-albumin; Prot-protein; PTH-parathyroid hormone; Cys C-Cystatin C.

We then compared the correlations of these 21 plasma biomarkers with clinical and laboratory parameters collected from patients with AKI (Fig. 3A). A wide variety of correlations were found in AKI patients between their plasma biomarker concentrations and demographic, hematological, hepatic, and chemical parameters. In terms of kidney function, β2-microglobulin, NGAL, Cystatin C, VEGF-A, renin and Calbindin all positively correlated with admission serum creatinine and peak creatinine. TFF3 also positively correlated with creatinine, and KIM-1 positively correlated with peak creatinine. Cystatin C, TFF3, VEGF-A and Renin positively correlated with serum urea. NGAL, cystatin C, VEGF-A, and renin positively correlated with total urine protein, and α1-microglobulin positively correlated with urine protein/creatinine ratio. Of all proteins, only β2-microglobulin positively correlated with initiation of dialysis in acute kidney disease. No significant correlations were found between the plasma biomarkers and either ICU or hospital length of stay. As all COVID-19 patients were on vasopressors, antibiotics, and steroids, and all patients were intubated, plasma correlations with these variables were forgone.

Correlation analyses were then performed between plasma biomarker levels from patients with CKD/ESKD and clinical and laboratory parameters (Fig. 3B). A wide variety of correlations were found in CKD/ESDR patients between their plasma biomarker concentrations and demographic, hematological, inflammatory, and chemical parameters. In terms of kidney function, β2-microglobulin, NGAL, Cystatin C, TFF3, α1-microglobulin, Uromodulin, and VEGF-A each positively correlated with creatinine and Cystatin C. TIMP-1 also positively correlated with Cystatin C, while β2-microglobulin and NGAL positively correlated with serum urea. TIMP-1, β2-microglobulin, NGAL, Cystatin C, TFF-3, α1-microglobulin, Uromodulin, VEGF-A, EGF, Clusterin, Osteoactivin and GSTA1 each negatively correlated with all kidney clearance measurements. IL-18 negatively correlated with CKD-EPI Creatinine and CKD-EPI Creatinine/Cystatin C clearance. KIM-1 negatively correlated with CKD-EPI Creatinine clearance measurement.

**Figure 4:**
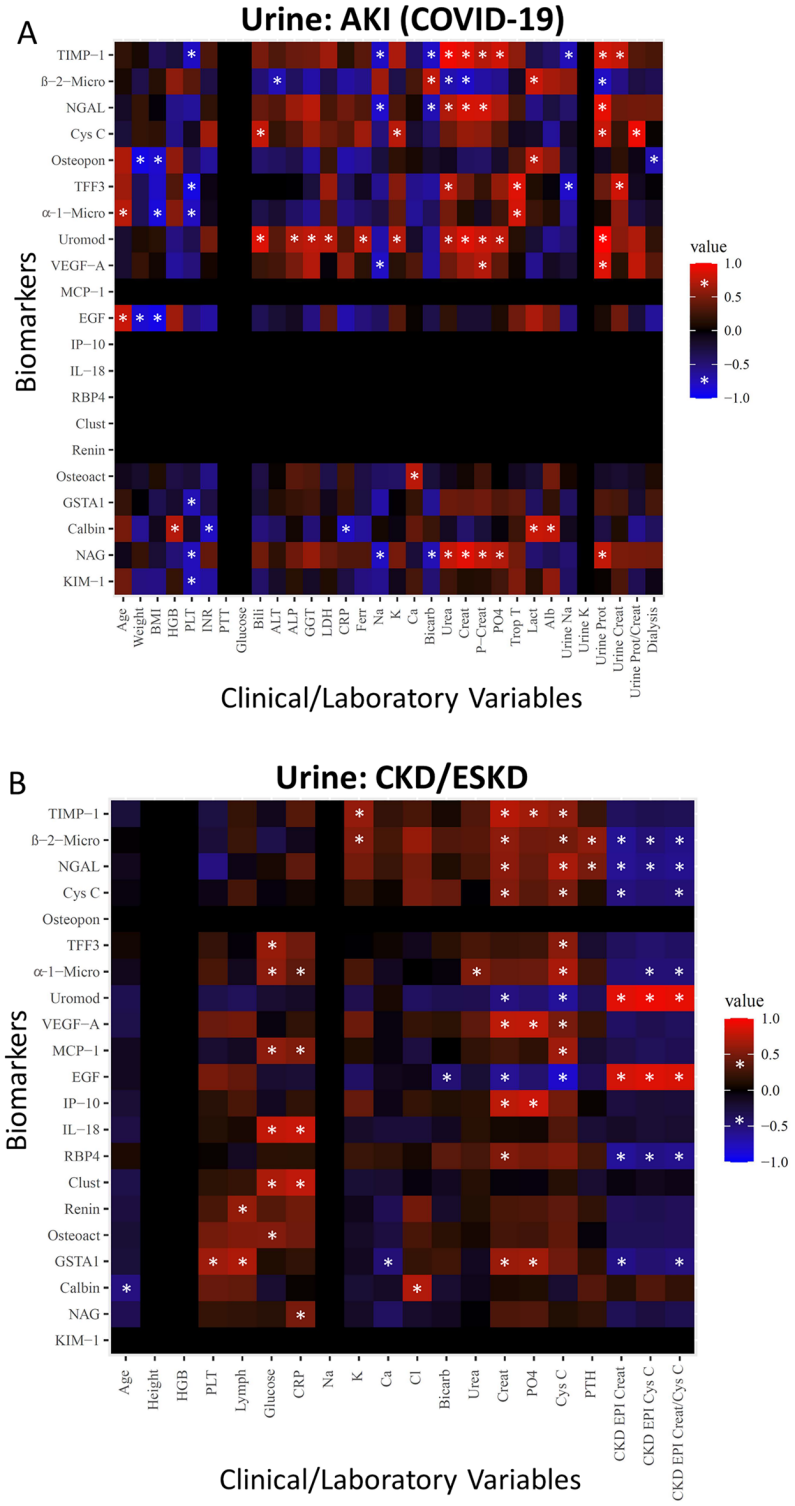
Correlations between urine biomarker expression and acute and chronic/end-stage kidney disease patient parameters. Heat maps of rank-based classifying proteins reported in Fig. 2 in acute kidney disease (**A**) and chronic/end-stage kidney disease (**B**) are illustrated (y -axis) along with patient parameters (x-axis). Only proteins that showed a significant correlation (*P* ≤ 0.05) with at least one parameter are illustrated. Significant correlations had a Pearson R-value of ≥ 0.7 or ≤ -0.7 for AKI, ≥ 0.4 or ≤ -0.4 for CKD/ESKD, and *p*-value < 0.05, denoted by *. Positive correlations are depicted in red and negative correlations in blue. Abbreviations: BMI-body mass index; HGB-hemoglobin; PLT-platelet; Lymph-lymphocytes; INR-international normalized ratio; PTT-partial thromboplastin time; Bili-total bilirubin; ALT-alanine aminotransferase; ALP-alkaline phosphatase; GGT-gamma glutamyl transferase; LDH-lactate dehydrogenase; CRP-C reactive protein; Ferr-ferritin; Na-sodium; K-potassium; Ca-calcium; Bicarb-bicarbonate; Cl-chloride; Creat-creatinine; P-Creat-peak creatinine; PO4-phosphate; Trop-troponin; Lact-lactate; Alb-albumin; Prot-protein; PTH-parathyroid hormone; Cys C-Cystatin C.

We then compared the correlations of these 21 urine biomarkers with clinical and laboratory parameters collected from patients with AKI (Fig. 4A). A wide variety of correlations were found in AKI patients between their plasma biomarker concentrations and demographic, hematological, hepatic, inflammatory and chemical parameters. From a kidney perspective, TIMP-1, NGAL, Uromodulin, and NAG each positively correlated with serum urea, creatinine, and peak creatinine. TFF3 positively correlated with serum urea, and VEGF-A positively correlated with peak creatine. β2-microglobulin negatively correlated with serum urea and creatinine. TIMP-1, NGAL, Cystatin C, Uromodulin, VEGF-A and NAG positively correlated with urine protein. β2-microglobulin negatively correlated with urine protein. TIMP-1 and TFF3 positively correlated with urine creatinine. Cystatin C solely positively correlated with urine protein/creatinine ratio, and Osteopontin solely negatively correlated with initiation of dialysis. No significant correlations were found between the urine biomarkers and either ICU or hospital length of stay. As all COVID-19 patients were on vasopressors, antibiotics, and steroids, and all patients were intubated, urine correlations with these variables were forgone.

Correlation analyses were then performed between urine biomarker levels from patients with CKD/ESKD and clinical and laboratory parameters (Fig. 4B). A wide variety of correlations were found in CKD/ESDR patients between their plasma biomarker concentrations and demographic, hematological, inflammatory, and chemical parameters. In terms of kidney function, TIMP-1, β2-microglobulin, NGAL, Cystatin C, VEGF-A positively correlated with serum creatinine and cystatin C. IP-10, RBP4 and GSTA1 also positively correlated with serum creatinine. TFF3, α1-microglobulin and MCP-1 positively correlated with serum Cystatin C while Uromodulin and EGF negatively correlated with serum cystatin C. Uromodulin and EGF also negatively correlated with serum creatinine. α1-microglobulin positively correlated with serum urea. β2-microglobulin, NGAL, and RBP4 each negatively correlated with all kidney clearance measurements. Uromodulin and EGF both positively correlated with all kidney clearance measurements. Cystatin C and GSTA1 negatively correlated with CKD-EPI Creatinine and CKD-EPI creatinine/cystatin C clearance. α1-microglobulin negatively correlated with CKD-EPI Cystatin C and CKD-EPI creatinine/cystatin C clearance.

As a final analysis, pairwise comparisons of the 21-biomarker profile between cohorts was achieved with Euclidian Distance and is shown in Supplementary Fig. 2. For both plasma and urine, healthy control subjects were relatively homogenous and distinct from patient cohorts.

## Discussion

Our study examined the plasma and urine profiles of 21 unique kidney toxicity biomarkers in patients with either AKI or CKD/ESKD. We reported differences in concentrations of these proteins, as well as their ability to differentiate patients with kidney disease from healthy control subjects. Biomarker rank order of importance was established. We also reported correlations of these biomarkers with patient demographic and clinical variables, including hematologic, hepatic, inflammatory, and chemical parameters.

Our patient cohort suffered either AKI or CKD/ESKD as defined by KDIGO classification. Hypertension and diabetes were the most common comorbidities in both acute and chronic disease. In fact, 50% of AKI patients had hypertension and diabetes. Moreover, all AKI patients had a presumed etiology related to tissue hypoperfusion and hemodynamic insult in the context of critical illness, in keeping with most causes of AKI in ICU patients^22^. With regard to CKD/ESKD, 85% had hypertension and 38% of CKD patients had diabetes; these findings are in keeping with major etiologies of CKD/ESKD worldwide^23^. Within the CKD/ESKD cohort, 9.5% of patients had CKD, 90.5% of patients had ESKD, with 76.1% being on intermittent hemodialysis, and 14.3% having kidney transplant.

Notably, when comparing plasma levels of biomarkers in healthy control subjects, and both AKI and CKD/ESKD patients, almost all protein concentrations differed significantly between the three groups; however, NAG and RBP4 were unchanged. When comparing urine biomarker levels, all proteins were significantly different in titer between the three cohorts, except Calbindin, Osteopontin and TIMP-1. These findings were consistent with reports of the 21 biomarkers having different roles in kidney injury^13^. β2-microglobulin, a low molecular weight protein that is used to assess tubular injury^24^, was among the most common highly ranked protein in plasma and urine that differentiated healthy control subjects from AKI and CKD/ESKD. β2-microglobulin has been a longstanding marker of kidney injury with increased urinary prevalence secondary to decreased tubule resorption post-injury^13^, with links to mortality in ESKD^25^ and AKI severity^26^.

When comparing plasma profiles of AKI versus CKD/ESKD, Osteopontin was the top ranked protein differentiating these populations. Osteopontin largely facilitates bone mineralization and resorption, but it is also present in the thick ascending limb and distal tubules^27^ where it mediates inflammation, angiogenesis, tubulogenesis, and apoptosis. Previous studies indicate that Osteopontin is elevated in AKI and CKD/ESKD, as well as kidney allograft dysfunction^27^. Similarly, urine α1-microglobulin was highly ranked in differentiating AKI versus CKD/ESKD patients. α1-microglobulin is a lipocalin filtered by the glomerulus, but fully reabsorbed by proximal tubular cells, suggesting urinary levels indicate tubular dysfunction. Urinary α1-microglobulin in HIV infected women is independently associated with kidney decline and mortality^28^. Together, Osteopontin and α1-microglobulin might serve biomarkers to characterize the different physiology underlying acute and chronic kidney disease.

Many biomarker levels correlated with demographic and clinical variables in both AKI and CKD/ESKD. Within the plasma of AKI patients, we examined correlations of biomarkers with hematologic, hepatic, inflammatory, and chemical variables. For hematology, Cystatin C, TFF3, and IL-18 negatively correlated with thrombocyte count. Although Cystatin C and TFF3 have weak links with thrombocyte physiology, IL-18 has been implicated in platelet activation and endothelial dysfunction^29^. Several markers, including β2-microglobulin, Osteopontin, α-1-microglobulin, VEGF-A, RBP4, Clusterin and Renin, positively correlated with the coagulation variables of INR or PTT. Urine β2-microglobulin titer has been linked with coagulation abnormalities in hemolytic-uremic syndrome^30^, and VEGF-A is associated with hypercoagulability in malignancy^31,32^. RBP4 is associated with inflammation and thrombogenesis in Kawasaki’s disease^33^, while renin–angiotensin–aldosterone activation is associated with atherothrombosis in COVID-19^34^.

In AKI patients, hepatic and inflammatory variables positively correlated with many plasma biomarkers. β2-microglobulin, NGAL and VEGF-A positively correlated with ALP and GGT, while VEGF-A, RBP4 and Clusterin positively correlated with ferritin. NGAL titers prognosticate survival in chronic liver disease^35^, and isoforms of VEGF are associated with hypertension and kidney dysfunction in non-alcoholic fatty liver disease^36^, as well as angiogenesis and inflammation^37^. RBP4 induces inflammation in endothelial cells^38^, and Clusterin may regulate inflammation via the NF-kβ pathway^39^. Uromodulin negatively correlated, and IP-10 positively correlated, with lactate, raising the question of their roles in mediating end-organ perfusion or dysfunction in AKI. Previous reports suggest Uromodulin predicts progression to ESKD^40^.

With electrolytes, most biomarker correlations were observed with phosphate. β2-microglobulin, NGAL, Cystatin C, TFF3, VEGF-A, IL-18, Renin, Calbindin and KIM-1 positively correlate with serum phosphate. Of these, only Calbindin has clear documentation of impacting electrolyte transport, impacting sodium-phosphate transport and cytoskeletal re-arrangement in experimental models of kidney tubular epithelial cells^41^.

Kidney variables correlated with plasma biomarkers. NGAL, Cystatin C, VEGF-A and Renin each correlated with admission creatinine, peak creatinine, and proteinuria, suggesting they may be heavily involved in pathogenesis of AKI. NGAL is produced by kidney tubular cells in response to insult, and it facilitates kidney development, tubular regeneration, and predicts AKI early in admission^42^. Cystatin C, is ubiquitously expressed by nucleated cells^43^ and it is a well-established kidney biomarker. Cystatine C levels positively correlated with VEGF-A, and may exert a protective effect in kidney injury with VEGF inhibition promoting proteinuria, hypertension and kidney injury^44,45^. The pathophysiology of VEGF in kidney disease is poorly elucidated, but may be related to endothelial cell proliferation, microvascular permeability, and matrix remodeling. VEGF is heavily expressed in glomerular podocytes and kidney tubular epithelial cells^46^. Renin, as part of the renin–angiotensin–aldosterone-system, mediates glomerular pressure as well as collecting duct solute transport^47^, with its blockade being extensively associated with improved kidney outcomes^48^.

In urine of AKI patients, TIMP-1 negatively correlated with platelet count. TIMP-1 is expressed by megakaryocytes and platelets to mediate tissue remodeling and angiogenesis^49^. With regard to hepatic function and inflammation, urinary Uromodulin emerged as the predominant biomarker that positively correlated with liver enzymes and ferritin. Decreased plasma Uromodulin is associated with kidney injury in cirrhotic patients^50^, yet our data suggested a positive correlation between urine Uromodulin and increasing liver enzymes. Plasma Uromodulin induces leukocyte recruitment in tubular injury and inflammation^51^, but little data exist on urinary uromodulin titers and inflammation.

More extensive urinary biomarker correlations were demonstrated with serum electrolytes in AKI, as compared to plasma. TIMP-1 and NAG negatively correlated with sodium and bicarbonate, and positively correlated with phosphate. NGAL negatively correlated with sodium and bicarbonate. Uromodulin positively correlated with potassium and phosphate. Notably, each of these biomarkers are heavily expressed in kidney tubule cells, perhaps explaining their association with electrolyte imbalance^52–54^.

Correlation analyses of kidney variables in AKI demonstrated that urine TIMP-1, NGAL, Uromodulin, and NAG positively correlated with admission urea and creatinine, peak creatinine, and urine protein, whereas the plasma biomarkers NGAL, Cystatin C, VEGF-A and Renin positively correlated with admission creatinine, peak creatinine and proteinuria. Urinary TIMP-1 predicts AKI in pediatric ICU patients^55^, and urinary NAG predicts kidney impairment in cystic fibrosis patients^56^. In contrast to our findings, urinary NGAL may be less useful to predict kidney injury in critically-ill septic patients^57^, and a systematic review has reported decreasing urine Uromodulin is associated with AKI^58^.

Distinct correlations were also observed in the plasma of CKD/ESKD patients. NGAL negatively correlated with hemoglobin, which is consistent with NGAL promoting anemia in inflammatory states^59^. Uromodulin positively correlated with lymphocyte count, which is in contrast of previous studies suggesting uromodulin inhibits lymphocyte proliferation^60^. Clusterin positively correlated with lymphocytes, with previous studies reporting an association between Clusterin and lymphoma pathogenesis^61^. α1-microglobulin positively correlated with platelet and lymphocyte count; the latter consistent with α1-microglobulin being actively produced by T and B cells^62^. Holistically, our correlations are in keeping with these biomarkers as possible regulators of blood cells in CKD, by inducing immune cell dysfunction and inflammation^63^.

From an inflammatory perspective, plasma Renin positively correlated with CRP, and ESKD is associated with inflammation predisposing to malignancy and infection^63^. Previous polymorphisms in the renin–angiotensin–aldosterone system pathway have been implicated in more rapid progression to ESKD; however, Renin itself has been less implicated^64,65^. Renin may mediate the inflammatory milieu in kidney disease and its contribution to the adverse cardiovascular outcomes noted in ESKD.

EGF negatively correlated with sodium in plasma from CKD/ESKD patients. EGF stimulates sodium resorption in alveolar epithelium^66^, but it has been unexplored in kidney electrolyte transport. Plasma Osteopontin positively correlated with potassium, consistent with potassium channel activation in pancreatic tissue^67^. β2-microglobulin negatively correlated with calcium. Experimental data suggest that β2-microglobulin may complex with calcium to facilitate amyloid deposition in tissue^68^ and β2-microglobulin levels rise in dialysis^69^, suggesting β2-microglobulin signaling as a potential target to modify the calcium dysregulation and amyloid deposition in ESKD. TIMP1, α1-microglobulin, Clusterin and Osteoactivin positively correlated with phosphate in CKD/ESKD plasma, as compared to AKI plasma. Certainty we note the limitations of these electrolyte data, given electrolyte levels will vary based on pre-selected dialysate targets, as well as fluctuations that occur in urine concentration.

From a kidney perspective, plasma levels of β2-microglobulin, NGAL, Cystatin C, TFF3, α1-microglobulin, Uromodulin and VEGF-A, positively correlated with pre-dialysis creatinine, and each negatively correlated with calculations of kidney clearance, consistent with each of these plasma biomarkers correlating with kidney impairment, similar to observations in AKI^13^. EGF, IL-18, Clusterin, Osteoactivin, GSTA1 and KIM-1 also negatively correlated with markers of clearance. Biomarker correlations with residual kidney clearance suggests that their associated signaling pathways may facilitate kidney recovery or preserve residual kidney function to improve quality of life. Pathway modulation could also help limit the cardiovascular, neurologic, and inflammatory sequelae associated with morbidity and mortality in ESKD. Osteopontin, NGAL, cystatin C, TFF3, TIMP1, and β2-microglobulin are upregulated in AKI post kidney transplant, with Osteopontin and TIMP-1 specifically upregulated in reversible injury compared to irreversible injury^70^.

Urine biomarkers in CKD/ESKD showed additional correlations with hematologic variables. GSTA1 positively correlated with platelets and lymphocytes, which are associations not previously reported. Moreover, Renin positively correlated with lymphocytes, consistent with reports of a unique lymphocyte population that may produce Renin to protect against infection, raising the question of whether this is an adaptive response that may occur in CKD/ESKD^71^.

Urine α1-microglobulin, MCP-1, IL-18, Clusterin and NAG positively correlated with serum CRP in CKD/ESKD patients. α1-microglobulin has been implicated in inflammatory bowel disease and hypertension^72,73^. MCP-1 is also known to mediate inflammation, and dysregulates glucose in acute myocardial infarction^74,75^. Of note, MCP-1 positively correlated with serum glucose in the CKD/ESKD patients in our study. IL-18 has been implicated in inflammatory kidney disease^76^. Clusterin deficiency has been associated with worsening kidney inflammation^77^.

With electrolytes, many of the correlations noted with the urine biomarkers we observe remain unelucidated (as described above for plasma) and may be of interest for further study. Interestingly, β2-microglobulin and NGAL positively correlated with parathyroid hormone (PTH), which is also unreported in the literature. Given issues with mineral bone disease in CKD/ESKD patients, these biomarkers may yield additional insight into PTH regulation.

In terms of CKD/ESKD kidney function, urine TIMP-1, β2-microglobulin, NGAL, cystatin C, VEGF-A, IP-10, RBP4 and GSTA1 positively correlated with creatinine, whereas Uromodulin and EGF negatively correlated with creatinine. The significance of this is limited given that most patients in this subgroup were on dialysis. β2-microglobulin, NGAL, α1-microglobulin, RBP4, and GSTA1 negatively correlated with kidney clearance. Notably the RB4 correlation was only observed in the urine of CKD/ESKD patients, unlike the other biomarkers that also occurred in the plasma of CKD/ESKD patients. Moreover, with urinary biomarkers, there were positive correlations with kidney clearance (unlike plasma biomarkers, which only negatively correlated with kidney clearance). Uromodulin and EGF positively correlated with residual kidney clearance, raising the question of protective effects and supported by reports of a negative association between urine Uromodulin and kidney injury^58^. EGF receptor activation is associated with kidney recovery in AKI, via epithelial cell regeneration^78^. Our data highlight the need for further investigating any kidney protective effects of EGF and Uromodulin in CKD/ESKD.

Our study has limitations. First, we recognize that not all clinical variables were similarly available or recorded in healthy control subjects and patients with either AKI or CKD/ESKD. Second, the number of AKI patients was limited, which may reduce the generalizability of the biomarkers. Third, all ESKD patients still produced urine in our study, suggesting results may not be generalizable to anuric ESKD patients. The utility of urinary biomarkers in anuric ESKD patients is questionable. Fourth, we recognize ESKD patients received dialysis, and hence correlations made with creatinine, electrolytes, and kidney clearance could have been impacted. However, several correlations in this population may still be useful to understanding physiology and adverse outcomes. Fifth, NAG was non-detectable in the majority of plasma samples; however, NAG is primarily located in the proximal tubular cells with urine levels are believed to originate exclusively in kidney. As the levels for NAG on the ProcartaPlex platform were below the lower limit of quantification in more than 95% of all samples irrespective of group, it was excluded in the final design of the Human ProcartaPlex™ Kidney Toxicity Panel 1 (EPX060-15857-901). Sixth, we did not normalize the urinary biomarkers to urinary concentration; normalization would lead to systematic bias due to conditions that characteristically have a larger impact on tubular function and concentrating ability. Finally, confounders not recorded, such as hypotension and volume status, may have impacted the biomarker profiles.

## Conclusions

In conclusion, this exploratory study characterized plasma and urine biomarker profiles in acute and chronic kidney disease. Utilizing machine learning and conventional statistics, we present novel profiles of biomarkers that differentiate healthy controls from kidney disease patients. A rank order of biomarker utility is provided, as well as accuracies. We report novel correlations of urine and plasma biomarkers with clinical/laboratory variables. Our findings highlight the ongoing need to investigate the interplay of these biomarkers with hematologic profiles, hepatic function, inflammation, electrolytes, and kidney function in acute and chronic kidney disease.

### Supplementary Information

Supplementary Information.

## Data Availability

The datasets generated and/or analyzed during the current study are available from the corresponding author on reasonable request.
