# Supplementary material for: A novel multiplex biomarker panel for profiling human acute and chronic kidney disease
Source: Sci Rep. 2023 Dec 1;13:21210. doi: 10.1038/s41598-023-47418-9 (PMC10692319; doi:10.1038/s41598-023-47418-9)
Supplement: Supplementary file 1 — Supplementary Information. [file 41598_2023_47418_MOESM1_ESM.docx]

**Supplementary Table 1. Function of 21 Renal Biomarkers.**

| **Biomarker** | **UniProt ID** | **Function** |
| --- | --- | --- |
| **KIM1** | **Q96D42** | 38.7 kDa Type I membrane protein with roles best characterized in renal disease, both acute and chronic. Heavily expressed in renal tissue. Elevated in response to interstitial diseases, polycystic kidney disease, and renal injury such as ischemia/reperfusion injury [1, 2]. Serum titres increase in response to stimuli via cleavage from the membrane by matrix metalloproteinases. Modulates NF-kB to inhibit proximal tubular cytokine release. Elevated levels in renal injury, diabetic nephropathy, transplant recipients, and medication induced renal damage [3]. Associated with renal fibrosis and inflammation. Modulates autophagy, DNA damage, renal vascular resistance, necrosis and apoptosis [4]. |
| **NAG** | **V9M3A9** | N-acetyl-glucosaminidase (NAG) is a lysosomal enzyme involved in hydrolysis of glycoprotein terminal glucose residues. Expressed primarily in the proximal tubular cells of the nephron. This largely facilitates degradation of cellular products, particularly cell membrane components. Urine excretion of NAG considered an early indicator for renal damage from nephrotoxic agents, diabetes, obstructive uropathy, hypertensive nephropathy [5, 6]. |
| **Calbindin** | **P05937** | A vitamin D-dependent calcium binding protein, involved in calcium regulation. Largely expressed in the distal convoluted tubule and collecting duct of the nephron, modulating calcium resorption [7]. |
| **GSTA1** | **P08263** | Glutathione S-transferase A1 is an enzyme with cytosolic and membrane bound forms. Regulates detoxification of electrophilic compounds via conjugation with nucleophilic reduced glutathione [8]. |
| **Osteoactivin** | **Q14956** | Type I transmembrane glycoprotein on plasma or endosome/lysosome membranes. Plays a role in differentiation of cell types including osteoblasts and osteoclasts. In the kidney heavily expressed in distal nephron, with urine titres increased in chronic and polycystic kidney disease, correlating with proteinuria, GFR, and serum creatinine [9]. |
| **Renin** | **P00797** | Aspartic protease protein secreted from the juxtaglomerular apparatus (in response to renal perfusion pressure) of the kidney, to initiate the renin-angiotensin-aldosterone (RAAS) cascade. RAAS system regulates renal glomerular pressure and sodium and potassium transport in the collecting duct [10]. Levels are associated with mortality and major adverse kidney events [11]. |
| **Clusterin** | **P10909** | 75-80KDa heat-shock protein expressed in epithelial and secretory cells in response to cellular stress [12]. Has both anti-apoptotic roles and protective roles in ischemia-reperfusion renal injury [13]. Deficiency in clusterin is associated with renal inflammation and fibrosis [14]. |
| **RBP4** | **P02753** | Retinol Binding Protein is a 21kDa plasma protein generated in the liver with affinity for lipophilic molecules involved in the transfer of vitamin A from hepatic stores to other tissues. Freely filtered by the renal glomeruli, and reabsorbed the proximal tubule, known to be associated with acute kidney injury [15-17]. |
| **IL-18** | **Q14116** | Member of the IL-1 superfamily. Promotes inflammation and the Th1 response modulating innate and adaptive immunity. Cleaved by casepase-1 to be released extracellularly from renal epithelial cells [18]. Upregulated in chronic renal disease and modulates renal fibrosis [19, 20]. |
| **IP-10** | **P02778** | 8.7 kDa is a small-inducible cytokine that is secreted by several cell types (monocytes, endothelial, fibroblasts) in response to [IFN-γ](https://en.wikipedia.org/wiki/Interferon_gamma). Binds to CXCR3 receptor to facilitate chemoattraction of monocytes, T cells, NJ cells, dendritic cells, inhibit bone marrow colony formation and angiogenesis [21]. Urinary excretion has been associated with post-transplant graft immune activation, acute rejection and impaired long-term graft function [22]. Blockade of IP-10 signalling contributes to renal fibrosis [23]. |
| **EGF** | **Q6QBS2** | Single polypeptide of 52 amino acid residues which promotes cell growth and differentiation via binding to EGFR. EGFR is expressed on a multitude of cell types, including the proximal and distal tubules of the kidney, and promotes tubular proliferation. Increased urinary titres of EGF are noted in diabetic nephropathy, lupus nephritis, IgA nephropathy and acute kidney injury [24]. Increased renal EGF activation has been implicated in the progression of renal disease [24] |
| **MCP-1** | **Q6UZ82** | Small cytokine belonging to the CC chemokine family, which directs monocytes, T-cells and dendritic cells to inflammation. Blockade of MCP-1 in renal disease models has minimized disease progression [25, 26]. Diabetes induced renal production of MCP-1 and urinary titres may associate with renal inflammation [27]. |
| **VEGF-A** | **P15692** | A member of the VEGF family which is a glycosylated mitogen which acts on endothelial cells to mediate vascular permeability, angiogenesis, cell migration and to inhibit apoptosis [28]. VEGF facilitates glomerular/tubular hypertrophy, and deficiency is associated with glomerulosclerosis and tubulointerstitial fibrosis, while increased titres may also be associated with chronic kidney disease [29]. |
| **Uromodulin** | **P07911** | Most abundant protein in human urine, generated only via renal tissue. Also known as Tamm-Horsfall protein. Largely generated in thick ascending limb of the loop of Henle. Positively correlates with glomerular filtration rate. Excreted into the urine following cleavage of the ectodomain of its glycophosphatidylinositol-anchored counterpart on the luminal surface of the loop of Henle [30, 31]. Appears to inhibit calcium crystallization in renal fluids and protective against urinary tract infections [31]. |
| **α-1-microglobulin** | **P02760** | Cellular housekeeping protein with reductase and radical binding activity. Synthesized in liver, freely filtered across the glomerulus then reabsorbed in proximal tubules. Urinary macroglobulin has been associated with AKI, CKD, cardiovascular events and mortality [32]. |
| **TFF3** | **Q07654** | Small peptide with roles in mucosal protection, cell proliferation and migration. Correlated with GI inflammation and solid tumors, increased titres in chronic kidney disease, and also associated with tubulointerstitial fibrosis [33]. Involved in glycan binding via cross-linking mucins to modulate thickness and viscosity of mucous, and activate PAR-2 receptors to induce cytokine regulation [34, 35]. |
| **Osteopontin** | **Q3LGBO** | Secreted acidic protein, rich in negatively charged aspartic and glutamic acid. Substrate for several enzymes promoting inhibition of bone mineralization to regulate bone remodelling. Also expressed in several immune cells with several immunomodulating properties [36]. Found in the distal renal tubule epithelial cells, loop of Henle, collecting duct and Bowman’s capsule, hypothesized to have roles in preventing apoptosis. Associated with renal outcome and survival in critically ill patients requiring renal replacement therapy [37]. |
| **Cystatin C** | **P01034** | 13 kDa endogenous cysteine proteinase inhibitor, ubiquity expressed on nucleated cells. 99% is freely filtered by glomeruli [38]. Extensive associated with acute kidney injury, glomerular filtration rate and all-cause and cardiovascular mortality [38]. |
| **NGAL** | **B2ZDQ1** | 25kDa protein of the lipocalin superfamily. Found in activated neutrophils, and renal tubular cells (particularly proximal tubule, and collecting ducts. Binds with siderophores to regulate iron metabolism. Involved in kidney development and renal tubular regeneration after injury [39]. High urinary levels associated with renal injury, renal non-recovery, in-hospital mortality, CKD progression and mortality [40]. |
| **β-2-microglobulin** | **A6XMH5** | 11.8 kDa protein located on the surface of nucleated cells; a component of Major Histocompatibility Class I molecules. Passed through glomeruli in kidneys then reabsorbed by the proximal tubule. Positive correlation between β2-microglobulin and mortality/morbidity in end stage renal disease [41]. Levels inversely correlate with glomerular filtration rate and positively with inflammation and malnutrition [42, 43]. |
| **TIMP1** | **P01033** | Metalloproteinase (MMP) inhibitor which irreversibly inhibits MMPs via binding to their catalytic zinc factor. Other roles in cell differentiation, migration and death via CD63 and ITGB1 signalling [44]. Increasing titres in blood and urine have correlated with chronic kidney disease, and may promote fibrosis [44, 45]. |

UniProt: The Universal Protein knowledgebase in 2023." *Nucleic Acids Research* 51, no. D1 (2023): D523-D531 [46]

**Supplementary Table 2. Plasma biomarker statistical comparisons.**

| **Protein** | **AKD vs Control**  **P-value** | **CKD/ESRD vs Control**  **P-value** | **CKD/ESRD vs AKD**  **P-value** |
| --- | --- | --- | --- |
| KIM-1 | **0.001** | 0.053 | 0.159 |
| NAG | 0.360 | 1.000 | 1.000 |
| Calbindin | **<.001** | 0.081 | **0.039** |
| GSTA1 | **<.001** | 0.141 | **0.030** |
| Osteoactivin | **0.009** | **0.007** | 1.000 |
| Renin | **<.001** | **0.013** | **0.024** |
| Clusterin | **0.004** | **0.024** | 0.710 |
| RBP4 | 0.080 | 0.378 | 0.765 |
| IL-18 | **<.001** | **0.013** | **0.017** |
| IP-10 | **<.001** | 0.062 | **0.002** |
| EGF | **0.001** | 0.113 | 0.064 |
| MCP-1 | **<.001** | **0.004** | 0.052 |
| VEGF-A | **0.011** | **<.001** | 1.000 |
| Uromodulin | 0.634 | **<.001** | 0.062 |
| α1-microglobulin | 1.000 | **0.001** | **<.001** |
| TFF3 | 0.145 | **<.001** | 0.112 |
| Osteopontin | **<.001** | **0.022** | **0.002** |
| Cystatin C | 0.175 | **<.001** | 0.138 |
| NGAL | 0.071 | **<.001** | 0.418 |
| β2-microglobulin | **0.019** | **<.001** | 0.945 |
| TIMP-1 | **<.001** | **0.007** | **0.004** |

**Supplementary Table 3: Urine biomarker statistical comparisons.**

| **Protein** | **AKD vs Control**  **P-value** | **CKD/ESRD vs Control**  **P-value** | **CKD/ESRD vs AKD**  **P-value** |
| --- | --- | --- | --- |
| KIM-1 | 1.000 | 0.055 | 0.138 |
| NAG | 1.000 | **0.047** | **0.047** |
| Calbindin | 0.432 | 0.806 | 1.000 |
| GSTA1 | 1.000 | **0.002** | **0.003** |
| Osteoactivin | 1.000 | **0.002** | **0.034** |
| Renin | 0.231 | **0.023** | **<.001** |
| Clusterin | 1.000 | **0.022** | 0.341 |
| RBP4 | 0.331 | **<.001** | 0.165 |
| IL-18 | 1.000 | 0.052 | **0.039** |
| IP-10 | 0.279 | 0.115 | **0.004** |
| EGF | 0.151 | **<.001** | 0.324 |
| MCP-1 | 1.000 | **0.001** | **0.020** |
| VEGF-A | 0.210 | **0.012** | 1.000 |
| Uromodulin | 0.075 | **0.002** | 0.983 |
| α1-microglobulin | **0.001** | **0.003** | 1.000 |
| TFF3 | 1.000 | **0.003** | **0.029** |
| Osteopontin | 0.330 | 0.213 | 1.000 |
| Cystatin C | 0.579 | **0.011** | 0.514 |
| NGAL | 0.371 | **0.002** | 0.359 |
| β2-microglobulin | 0.302 | **<.001** | 0.190 |
| TIMP-1 | 0.789 | 0.398 | 1.000 |

**Supplementary Table 4: Week 1 Pre-Post ESRD dialysis plasma biomarkers.**

***values are mean (standard deviation)**

| **Protein** | **Pre-dialysis**  **(n=3)** | **Post-dialysis**  **(n=3)** | **P-value** |
| --- | --- | --- | --- |
| KIM-1 | 322.4 (67.6) | 425.0 (109.8) | 0.109 |
| NAG | 0 (0-0) | 276.0 (356.6) | 0.180 |
| Calbindin | 24.0 (14.9) | 52.6 (21.3) | 0.109 |
| GSTA1 | 255.4 (83.5) | 247.4 (66.2) | >0.994 |
| Osteoactivin | 433.0 (90.5) | 538.9 (441.4) | >0.994 |
| Renin | 87.0 (63.8) | 117.0 (126.1) | >0.994 |
| Clusterin | 570261 (173712) | 432561 (160813) | 0.109 |
| RBP4 | 113119 (109092) | 86284.0 (61693) | 0.593 |
| IL-18 | 37.8 (12.2) | 34.7 (3.7) | 0.593 |
| IP-10 | 10.5 (6.6) | 8.2 (2.1) | 0.593 |
| EGF | 2.4 (1.1) | 4.0 (3.3) | 0.593 |
| MCP-1 | 24.3 (7.4) | 36.2 (34.2) | >0.994 |
| VEGF-A | 8110 (138.5) | 662.0 (361.5) | 0.285 |
| Uromodulin | 1342 (485) | 1716 (647) | 0.109 |
| α1-microglobulin | 6238 (936) | 8513 (3688) | 0.285 |
| TFF3 | 73.7 (19.4) | 37.3 (3.2) | 0.109 |
| Osteopontin | 71.1 (16.4) | 86.1 (14.0) | 0.109 |
| Cystatin C | 589.2 (88.6) | 584.3 (234.7) | >0.994 |
| NGAL | 648.5 (62.6) | 608.5 (103.3) | 0.109 |
| β2-microglobulin | 7234 (1996) | 6658 (3574) | 0.593 |
| TIMP-1 | 97.5 (28.5) | 101.1 (53.8) | 0.593 |

**Supplementary Table 5: Week 14 ESRD Pre-Post dialysis plasma biomarkers.**

***values are mean (standard deviation)**

| **Protein** | **Pre-dialysis**  **(n=3)** | **Post-dialysis**  **(n=3)** | **P-value** |
| --- | --- | --- | --- |
| KIM-1 | 361.7 (12.8) | 361.7 (12.83) | 0.593 |
| NAG | 0 (0-0) | 0 (0-0) | >0.994 |
| Calbindin | 32.1 (11.6) | 32.1 (11.6) | >0.994 |
| GSTA1 | 321.2 (36.7) | 321.2 (36.7) | 0.593 |
| Osteoactivin | 337.2 (103) | 337.2 (103.0) | 0.109 |
| Renin | 84.1 (71.1) | 84.1 (71.1) | 0.109 |
| Clusterin | 509694 (119455) | 509694 (119455) | 0.109 |
| RBP4 | 99500 (28524) | 99500 (28524) | 0.285 |
| IL-18 | 37.4 (6.7) | 37.4 (6.7) | 0.593 |
| IP-10 | 5.2 (2.6) | 5.2 (2.6) | 0.109 |
| EGF | 3.7 (1.0) | 4.4 (0) | 0.109 |
| MCP-1 | 20.6 (4.9) | 20.6 (4.9) | 0.109 |
| VEGF-A | 219.7 (74.8) | 219.7 (74.8) | 0.109 |
| Uromodulin | 1282 (661) | 1282 (661) | >0.994 |
| α1-microglobulin | 6128 (1474) | 6128 (1474) | 0.285 |
| TFF3 | 21.6 (4.1) | 21.6 (4.1) | 0.109 |
| Osteopontin | 81.7 (27.5) | 81.7 (27.5) | 0.109 |
| Cystatin C | 337.2 (41.1) | 337.2 (41.1) | 0.109 |
| NGAL | 358.3 (43.8) | 358.3 (43.8) | 0.109 |
| β2-microglobulin | 3886 (1026) | 3886 (1026) | 0.109 |
| TIMP-1 | 67.3 (31.1) | 67.3 (31.1) | 0.109 |

**Supplementary Table 6: Plasma Biomarker Logistic Regression ROC curve, F1, PPV, NPV, and P-value.**

| **Protein** | **AKI (COVID-19) vs Control** | | | | |
| --- | --- | --- | --- | --- | --- |
|  | **ROC curve AUC (95%CI)** | **F1** | **PPV** | **NPV** | **P-value** |
| KIM-1 | 1.00 (1.00-1.00) | 1.00 | 1.00 | 1.00 | **0.003** |
| NAG | 0.60 (0.59-0.61) | 0.32 | 0.43 | 0.45 | 1.000 |
| Calbindin | 0.99 (0.98-0.99) | 0.90 | 0.92 | 0.93 | **0.026** |
| GSTA1 | 1.00 (1.00-1.00) | 0.96 | 0.97 | 0.97 | **0.003** |
| Osteoactivin | 0.91 (0.91-0.92) | 0.75 | 0.81 | 0.82 | 0.062 |
| Renin | 1.00 (1.00-1.00) | 0.99 | 1.00 | 0.99 | **0.003** |
| Clusterin | 0.94 (0.93-0.94) | 0.66 | 0.50 | 0.50 | **0.039** |
| RBP4 | 0.50 (0.48-0.52) | 0.32 | 0.24 | 0.27 | 0.434 |
| IL-18 | 1.00 (1.00-1.00) | 0.96 | 1.00 | 0.96 | **0.003** |
| IP-10 | 1.00 (1.00-1.00) | 0.96 | 1.00 | 0.95 | **0.003** |
| EGF | 1.00 (1.00-1.00) | 0.99 | 0.98 | 1.00 | **0.019** |
| MCP-1 | 1.00 (1.00-1.00) | 0.92 | 0.96 | 0.93 | **0.003** |
| VEGF-A | 1.00 (1.00-1.00) | 0.99 | 1.00 | 0.99 | **0.003** |
| Uromodulin | 0.73 (0.72-0.74) | 0.64 | 0.74 | 0.69 | 1.000 |
| α1-microglobulin | 0.64 (0.62-0.65) | 0.53 | 0.56 | 0.54 | 1.000 |
| TFF3 | 0.98 (0.98-0.99) | 0.89 | 0.96 | 0.90 | **0.007** |
| Osteopontin | 1.00 (1.00-1.00) | 1.00 | 1.00 | 1.00 | **0.003** |
| Cystatin C | 0.90 (0.89-0.91) | 0.76 | 0.82 | 0.80 | 0.098 |
| NGAL | 0.95 (0.95-0.96) | 0.87 | 0.92 | 0.89 | **0.023** |
| β2-microglobulin | 1.00 (1.00-1.00) | 0.98 | 0.99 | 0.98 | **0.003** |
| TIMP-1 | 1.00 (1.00-1.00) | 1.00 | 1.00 | 1.00 | **0.003** |

* p-value – Bonferroni Corrected Mann-Whitney

**Supplementary Table 7: Plasma Biomarker Logistic Regression ROC curve, F1, PPV, NPV, and P-value.**

| **Protein** | **CKD/ESKD vs Control** | | | | |
| --- | --- | --- | --- | --- | --- |
|  | **ROC curve AUC (95%CI)** | **F1** | **PPV** | **NPV** | **P-value** |
| KIM-1 | 0.80 (0.80-0.81) | 0.80 | 0.79 | 0.38 | 0.282 |
| NAG | 0.51 (0.51-0.51) | 0.84 | 0.73 | 0.00 | 1.000 |
| Calbindin | 0.77 (0.76-0.77) | 0.81 | 0.80 | 0.32 | 0.577 |
| GSTA1 | 0.76 (0.76-0.77) | 0.80 | 0.77 | 0.26 | 0.666 |
| Osteoactivin | 0.86 (0.85-0.87) | 0.89 | 0.87 | 0.61 | **0.026** |
| Renin | 0.91 (0.91-0.91) | 0.88 | 0.90 | 0.68 | **0.006** |
| Clusterin | 0.83 (0.83-0.84) | 0.84 | 0.73 | 0.00 | 0.104 |
| RBP4 | 0.50 (0.49-0.52) | 0.40 | 0.35 | 0.14 | 1.000 |
| IL-18 | 0.92 (0.91-0.92) | 0.89 | 0.91 | 0.73 | **0.015** |
| IP-10 | 0.85 (0.84-0.86) | 0.88 | 0.86 | 0.60 | **0.048** |
| EGF | 0.77 (0.77-0.78) | 0.80 | 0.79 | 0.33 | 0.586 |
| MCP-1 | 0.96 (0.96-0.96) | 0.93 | 0.94 | 0.86 | **<0.001** |
| VEGF-A | 0.94 (0.94-0.95) | 0.93 | 0.98 | 0.80 | **0.001** |
| Uromodulin | 0.97 (0.97-0.97) | 0.92 | 0.94 | 0.82 | **0.003** |
| α1-microglobulin | 0.96 (0.96-0.97) | 0.92 | 0.94 | 0.81 | **<0.001** |
| TFF3 | 0.97 (0.96-0.97) | 0.96 | 0.99 | 0.89 | **0.003** |
| Osteopontin | 0.92 (0.91-0.92) | 0.89 | 0.91 | 0.73 | **0.005** |
| Cystatin C | 0.97 (0.97-0.97) | 0.94 | 0.96 | 0.84 | **<0.001** |
| NGAL | 0.96 (0.96-0.96) | 0.95 | 0.97 | 0.87 | **<0.001** |
| β2-microglobulin | 0.98 (0.98-0.98) | 0.96 | 0.98 | 0.88 | **<0.001** |
| TIMP-1 | 0.97 (0.97-0.97) | 0.93 | 0.96 | 0.84 | **<0.001** |

* p-value – Bonferroni Corrected Mann-Whitney

**Supplementary Table 8: Urine Biomarker Logistic Regression ROC curve, F1, PPV, NPV, and P-value.**

| **Protein** | **AKI (COVID-19) vs Control** | | | | |
| --- | --- | --- | --- | --- | --- |
|  | **ROC curve AUC (95%CI)** | **F1** | **PPV** | **NPV** | **P-value** |
| KIM-1 | 0.74 (0.73-0.75) | 0.58 | 0.67 | 0.65 | 1.000 |
| NAG | 0.88 (0.87-0.88) | 0.78 | 0.94 | 0.81 | 0.096 |
| Calbindin | 0.52 (0.51-0.54) | 0.44 | 0.43 | 0.39 | 1.000 |
| GSTA1 | 1.00 (1.00-1.00) | 0.95 | 0.99 | 0.95 | **0.020** |
| Osteoactivin | 1.00 (1.00-1.00) | 0.93 | 0.95 | 0.95 | **0.003** |
| Renin | 0.94 (0.93-0.94) | 0.86 | 0.94 | 0.87 | **0.039** |
| Clusterin | 0.71 (0.70-0.73) | 0.64 | 0.49 | 0.03 | 1.000 |
| RBP4 | 0.86 (0.85-0.87) | 0.75 | 0.80 | 0.64 | 0.310 |
| IL-18 | 0.79 (0.78-0.80) | 0.65 | 0.73 | 0.70 | 0.796 |
| IP-10 | 0.87 (0.86-0.88) | 0.81 | 0.89 | 0.84 | 0.219 |
| EGF | 0.82 (0.81-0.83) | 0.51 | 0.54 | 0.64 | 0.591 |
| MCP-1 | 0.87 (0.86-0.88) | 0.74 | 0.83 | 0.78 | 0.219 |
| VEGF-A | 0.59 (0.58-0.60) | 0.49 | 0.57 | 0.51 | 1.000 |
| Uromodulin | 0.62 (0.61-0.64) | 0.11 | 0.09 | 0.46 | 1.000 |
| α1-microglobulin | 0.49 (0.48-0.50) | 0.44 | 0.48 | 0.45 | 1.000 |
| TFF3 | 0.77 (0.76-0.78) | 0.80 | 0.92 | 0.82 | 1.000 |
| Osteopontin | 0.49 (0.48-0.51) | 0.43 | 0.48 | 0.44 | 1.000 |
| Cystatin C | 0.64 (0.63-0.65) | 0.55 | 0.75 | 0.65 | 1.000 |
| NGAL | 0.76 (0.75-0.77) | 0.66 | 0.77 | 0.72 | 1.000 |
| β2-microglobulin | 0.86 (0.85-0.86) | 0.81 | 0.94 | 0.82 | 0.310 |
| TIMP-1 | 0.49 (0.48-0.51) | 0.44 | 0.46 | 0.41 | 1.000 |

* p-value – Bonferroni Corrected Mann-Whitney

**Supplementary Table 9: Urine Biomarker Logistic Regression ROC curve, F1, PPV, NPV, and P-value.**

| **Protein** | **CKD/ESKD vs Control** | | | | |
| --- | --- | --- | --- | --- | --- |
|  | **ROC curve AUC (95%CI)** | **F1** | **PPV** | **NPV** | **P-value** |
| KIM-1 | 0.81 (0.80-0.81) | 0.82 | 0.80 | 0.47 | 0.279 |
| NAG | 0.73 (0.72-0.73) | 0.78 | 0.74 | 0.08 | 0.679 |
| Calbindin | 0.58 (0.57-0.59) | 0.79 | 0.71 | 0.19 | 1.000 |
| GSTA1 | 0.92 (0.92-0.93) | 0.85 | 0.90 | 0.72 | **0.016** |
| Osteoactivin | 0.86 (0.86-0.87) | 0.83 | 0.89 | 0.61 | 0.056 |
| Renin | 0.88 (0.88-0.89) | 0.83 | 0.87 | 0.65 | **0.028** |
| Clusterin | 0.83 (0.83-0.84) | 0.82 | 0.70 | 0.02 | 0.132 |
| RBP4 | 0.94 (0.93-0.94) | 0.87 | 0.88 | 0.58 | **0.009** |
| IL-18 | 0.79 (0.78-0.80) | 0.83 | 0.79 | 0.40 | 0.194 |
| IP-10 | 0.79 (0.78-0.80) | 0.81 | 0.79 | 0.42 | 0.332 |
| EGF | 0.94 (0.93-0.94) | 0.88 | 0.89 | 0.74 | **0.003** |
| MCP-1 | 0.95 (0.95-0.95) | 0.88 | 0.92 | 0.75 | **0.001** |
| VEGF-A | 0.85 (0.84-0.86) | 0.82 | 0.84 | 0.51 | 0.070 |
| Uromodulin | 0.90 (0.89-0.91) | 0.36 | 0.36 | 0.45 | **0.005** |
| α1-microglobulin | 0.94 (0.94-0.95) | 0.89 | 0.93 | 0.78 | **0.009** |
| TFF3 | 0.96 (0.95-0.96) | 0.92 | 0.95 | 0.85 | **0.001** |
| Osteopontin | 0.72 (0.71-0.73) | 0.82 | 0.76 | 0.37 | 1.000 |
| Cystatin C | 0.88 (0.88-0.88) | 0.84 | 0.92 | 0.66 | **0.028** |
| NGAL | 0.90 (0.90-0.91) | 0.88 | 0.95 | 0.75 | **0.012** |
| β2-microglobulin | 0.94 (0.93-0.94) | 0.90 | 0.96 | 0.78 | **0.003** |
| TIMP-1 | 0.64 (0.63-0.65) | 0.78 | 0.72 | 0.16 | 1.000 |

* p-value – Bonferroni Corrected Mann-Whitney

**Supplementary Table 10: Classification Ability of the 21 Biomarkers**

|  | **Balanced Accuracy** | **ROC AUC** | **F1** | **Precision** | **Recall** |
| --- | --- | --- | --- | --- | --- |
| **Plasma** |  |  |  |  |  |
| Healthy Controls vs AKI (COVID-19) | 1.00 | 1.00 | 1.00 | 1.00 | 1.00 |
| Healthy Controls vs CKD/ESKD | 0.90 | 0.93 | 0.92 | 0.96 | 0.90 |
| AKI (COVID-19) vs CKD/ESKD | 0.95 | 1.00 | 0.95 | 1.00 | 0.90 |
| Healthy Controls vs AKI (COVID-19) vs CKD/ESKD * | 0.89 | 0.97 | -- | -- | -- |
| **Urine** |  |  |  |  |  |
| Healthy Controls vs AKI (COVID-19) | 0.69 | 0.94 | 0.55 | 1.00 | 0.39 |
| Healthy Controls vs CKD/ESKD | 0.83 | 0.94 | 0.83 | 0.95 | 0.78 |
| AKI (COVID-19) vs CKD/ESKD | 0.5 | 0.60 | 0.63 | 0.74 | 0.67 |
| Healthy Controls vs AKI (COVID-19) vs CKD/ESKD * | 0.67 | 0.88 | -- | -- | -- |

* Balanced accuracy not applicable, normal accuracy used. ROC AUC one-vs-one weighted used to mitigate class-imbalance.

| **Protein** | **ESRD**  **(4 patients)** | **ESRD**  **(17 patients)** | **P-value** |
| --- | --- | --- | --- |
| Clusterin | 260274.3 (240254.6-275389.1) | 531462.1 (399209.2-691048.4) | **0.007** |
| VEGF-A | 55.0 (38.2-93.8) | 722.6 (599.4-817.8) | **0.007** |
| TIMP-1 | 50.4 (41.2-58.8) | 92.4 (85.5-99.4) | **0.007** |
| Osteoactivin | 237.9 (198.1-282.9) | 581.1 (455.8-892.7) | **0.007** |
| β2-microglobulin | 2109.8 (1401.5-2766.5) | 5307.6 (4413.3-5982.6) | **0.014** |
| TFF3 | 9.5 (7.4-13.5) | 58.2 (48.1-62.5) | **0.028** |
| Cystatin C | 321.1 (239.5-392.3) | 625.1 (542.5-706.3) | **0.028** |
| α1-microglobulin | 1400.4 (1153.2-1794.8) | 4651.5 (4096.0-5236.3) | **0.049** |
| Uromodulin | 319.4 (194.1-429.2) | 1432.7 (1131.7-1706.4) | 0.056 |
| GSTA1 | 152.6 (114.9-187.0) | 486.3 (348.2-545.4) | 0.084 |
| Calbindin | 0.0 (0.0-0.0) | 48.0 (31.4-71.4) | 0.094 |
| KIM-1 | 233.2 (203.7-266.4) | 651.6 (393.6-790.8) | 0.126 |
| MCP-1 | 18.7 (17.4-19.7) | 43.6 (31.4-66.7) | 0.126 |
| Il-18 | 33.6 (27.3-37.4) | 94.9 (49.4-110.0) | 0.172 |
| EGF | 0.8 (0.6-1.4) | 14.7 (5.5-18.9) | 0.196 |
| NGAL | 183.6 (124.5-250.4) | 380.5 (313.7-604.9) | 0.266 |
| Osteopontin | 35.3 (26.9-46.2) | 59.9 (37.9-86.5) | 1.000 |
| RBP4 | 82696.3 (75941.4-92546.3) | 236390.9 (86413.4-355201.8) | 1.000 |
| Renin | 104.4 (104.3-153.9) | 164.8 (94.8-351.2) | 1.000 |
| NAG | 0.0 (0.0-0.0) | 0.0 (0.0-0.0) | 1.000 |
| IP-10 | 6.7 (5.2-8.8) | 12.6 (8.0-18.2) | 1.000 |

**Supplementary Table 11: CKD/ESRD Subgroup Biomarker Comparisons.**

**References:**

1. Huang, Y. and A. Craig Don-Wauchope, The clinical utility of kidney injury molecule 1 in the prediction, diagnosis and prognosis of acute kidney injury: a systematic review. Inflammation & Allergy-Drug Targets (Formerly Current Drug Targets-Inflammation & Allergy)(Discontinued), 2011. 10(4): p. 260-271.

2. Bonventre, J.V., Kidney injury molecule-1 (KIM-1): a urinary biomarker and much more. 2009, Oxford University Press. p. 3265-3268.

3. Van Timmeren, M.M., et al., High urinary excretion of kidney injury molecule-1 is an independent predictor of graft loss in renal transplant recipients. Transplantation, 2007. 84(12): p. 1625.

4. Han, W.K., et al., Kidney Injury Molecule-1 (KIM-1): a novel biomarker for human renal proximal tubule injury. Kidney international, 2002. 62(1): p. 237-244.

5. Kim, S.R., et al., Urinary N-acetyl-β-D-glucosaminidase, an early marker of diabetic kidney disease, might reflect glucose excursion in patients with type 2 diabetes. Medicine, 2016. 95(27).

6. Liangos, O., et al., Urinary N-acetyl-β-(D)-glucosaminidase activity and kidney injury molecule-1 level are associated with adverse outcomes in acute renal failure. Journal of the American Society of Nephrology, 2007. 18(3): p. 904-912.

7. Lee, C.-T., et al., The role of calbindin-D28k on renal calcium and magnesium handling during treatment with loop and thiazide diuretics. American Journal of Physiology-Renal Physiology, 2016. 310(3): p. F230-F236.

8. Economopoulos, K.P. and T.N. Sergentanis, GSTM1, GSTT1, GSTP1, GSTA1 and colorectal cancer risk: a comprehensive meta-analysis. European journal of cancer, 2010. 46(9): p. 1617-1631.

9. Pahl, M.V., et al., Upregulation of monocyte/macrophage HGFIN (Gpnmb/Osteoactivin) expression in end-stage renal disease. Clinical Journal of the American Society of Nephrology, 2010. 5(1): p. 56-61.

10. Brewster, U.C. and M.A. Perazella, The renin-angiotensin-aldosterone system and the kidney: effects on kidney disease. The American journal of medicine, 2004. 116(4): p. 263-272.

11. Siew, E.D., et al., Renin-angiotensin aldosterone inhibitor use at hospital discharge among patients with moderate to severe acute kidney injury and its association with recurrent acute kidney injury and mortality. Kidney International, 2021. 99(5): p. 1202-1212.

12. Jones, S.E. and C. Jomary, Clusterin. The international journal of biochemistry & cell biology, 2002. 34(5): p. 427-431.

13. Dieterle, F., et al., Urinary clusterin, cystatin C, β2-microglobulin and total protein as markers to detect drug-induced kidney injury. Nature biotechnology, 2010. 28(5): p. 463-469.

14. Guo, J., et al., Relationship of clusterin with renal inflammation and fibrosis after the recovery phase of ischemia-reperfusion injury. BMC nephrology, 2016. 17(1): p. 1-15.

15. Xun, C., et al., Circulating RBP4 increase and its diagnosis of chronic kidney disease. Annals of Clinical & Laboratory Science, 2018. 48(2): p. 205-207.

16. Zhang, L., et al., The role of circulating RBP4 in the type 2 diabetes patients with kidney diseases: a systematic review and meta-analysis. Disease Markers, 2020. 2020.

17. Ratajczyk, K., et al., The Clinical Significance of Urinary Retinol-Binding Protein 4: A Review. International Journal of Environmental Research and Public Health, 2022. 19(16): p. 9878.

18. Hirooka, Y. and Y. Nozaki, Interleukin-18 in inflammatory kidney disease. Frontiers in medicine, 2021. 8: p. 639103.

19. Araki, S., et al., Predictive impact of elevated serum level of IL-18 for early renal dysfunction in type 2 diabetes: an observational follow-up study. Diabetologia, 2007. 50: p. 867-873.

20. Parikh, C.R. and P. Devarajan, New biomarkers of acute kidney injury. Critical care medicine, 2008. 36(4): p. S159-S165.

21. Cassatella, M.A., et al., Regulated production of the interferon‐γ‐inducible protein− 10 (IP‐10) chemokine by human neutrophils. European journal of immunology, 1997. 27(1): p. 111-115.

22. Matz, M., et al., Early post-transplant urinary IP-10 expression after kidney transplantation is predictive of short-and long-term graft function. Kidney international, 2006. 69(9): p. 1683-1690.

23. Nakaya, I., et al., Blockade of IP-10/CXCR3 promotes progressive renal fibrosis. Nephron Experimental nephrology, 2007. 107(1): p. e12-e21.

24. Harskamp, L.R., et al., The epidermal growth factor receptor pathway in chronic kidney diseases. Nature Reviews Nephrology, 2016. 12(8): p. 496-506.

25. Giunti, S., et al., Targeting the MCP-1/CCR2 system in diabetic kidney disease. Current vascular pharmacology, 2010. 8(6): p. 849-860.

26. Munshi, R., et al., MCP-1 gene activation marks acute kidney injury. Journal of the American Society of Nephrology, 2011. 22(1): p. 165-175.

27. Tesch, G.H., MCP-1/CCL2: a new diagnostic marker and therapeutic target for progressive renal injury in diabetic nephropathy. American Journal of Physiology-Renal Physiology, 2008. 294(4): p. F697-F701.

28. Neufeld, G., et al., Vascular endothelial growth factor (VEGF) and its receptors. The FASEB journal, 1999. 13(1): p. 9-22.

29. Majumder, S. and A. Advani, VEGF and the diabetic kidney: more than too much of a good thing. Journal of Diabetes and its Complications, 2017. 31(1): p. 273-279.

30. Lhotta, K., Uromodulin and chronic kidney disease. Kidney and Blood Pressure Research, 2010. 33(5): p. 393-398.

31. El-Achkar, T.M., et al., Tamm-Horsfall protein translocates to the basolateral domain of thick ascending limbs, interstitium, and circulation during recovery from acute kidney injury. American Journal of Physiology-Renal Physiology, 2013. 304(8): p. F1066-F1075.

32. Robles, N.R., et al., Alpha-1-microglobulin: Prognostic value in chronic kidney disease. Medicina Clínica (English Edition), 2021. 157(8): p. 368-370.

33. Im, S., et al., Reduced expression of TFF1 and increased expression of TFF3 in gastric cancer: correlation with clinicopathological parameters and prognosis. International journal of medical sciences, 2013. 10(2): p. 133.

34. Du, T.-y., et al., Circulating serum trefoil factor 3 (TFF3) is dramatically increased in chronic kidney disease. PloS one, 2013. 8(11): p. e80271.

35. Lin, Z., et al., Trefoil factor 3: New highlights in chronic kidney disease research. Cellular Signalling, 2022: p. 110470.

36. Varalakshmi, B., et al., Plasma osteopontin levels in patients with acute kidney injury requiring dialysis: a study in a tertiary care institute in South India. International Urology and Nephrology, 2020. 52: p. 917-921.

37. Lorenzen, J.M., et al., Osteopontin predicts survival in critically ill patients with acute kidney injury. Nephrology Dialysis Transplantation, 2011. 26(2): p. 531-537.

38. Dharnidharka, V.R., C. Kwon, and G. Stevens, Serum cystatin C is superior to serum creatinine as a marker of kidney function: a meta-analysis. American journal of kidney diseases, 2002. 40(2): p. 221-226.

39. Bolignano, D., et al., Neutrophil gelatinase–associated lipocalin (NGAL) as a marker of kidney damage. American journal of kidney diseases, 2008. 52(3): p. 595-605.

40. McMahon, B.A., et al., Biomarker predictors of adverse acute kidney injury outcomes in critically ill patients: The Dublin acute biomarker group evaluation study. American Journal of Nephrology, 2019. 50(1): p. 19-28.

41. Puthiyottil, D., et al., Role of urinary beta 2 microglobulin and kidney injury molecule-1 in predicting kidney function at one year following acute kidney injury. International Journal of Nephrology and Renovascular Disease, 2021: p. 225-234.

42. Wu, H.C., L.C. Lee, and W.J. Wang, Associations among serum beta 2 microglobulin, malnutrition, inflammation, and advanced cardiovascular event in patients with chronic kidney disease. Journal of clinical laboratory analysis, 2017. 31(3): p. e22056.

43. Argyropoulos, C.P., et al., Rediscovering beta-2 microglobulin as a biomarker across the spectrum of kidney diseases. Frontiers in medicine, 2017. 4: p. 73.

44. Ries, C., Cytokine functions of TIMP-1. Cellular and molecular life sciences, 2014. 71: p. 659-672.

45. Bojic, S., et al., Diagnostic value of matrix metalloproteinase-9 and tissue inhibitor of matrix metalloproteinase-1 in sepsis-associated acute kidney injury. The Tohoku Journal of Experimental Medicine, 2015. 237(2): p. 103-109.

46. UniProt: The Universal Protein knowledgebase in 2023. Nucleic Acids Research, 2023. 51(D1): p. D523-D531.

**SUPPLEMENTARY FIGURE LEGEND**

**Supplementary Figure 1.** **Plasma and urine biomarkers accurately differentiate acute kidney disease and chronic/end-stage renal disease.** In the upper section, t-SNE plots depict the separation between acute kidney injury (AKI; blue dots) patients from chronic/end-stage renal disease (CKD/ESRD; red dots) patients by concentrations of plasma (A) and urine (C) biomarkers. Subjects plotted in 2D following dimensionality reduction of their respective proteomes by stochastic neighbour embedding. Axes are dimensionless. The dimensionality reduction shows that based on plasma or urine proteome, the two cohorts are reasonably distinct and separable A) Blue dots represent AKI patients and pink dots represent CKD/ESRD. B) Plasma biomarkers that distinguished AKI from CKD/ESRD patients in order of importance. D) Urine biomarkers that distinguished AKI from CKD/ESRD patients in order of importance.

**Supplementary Figure 2.** **Plasma and urine biomarker profiles compared pairwise to all patients.** Lower distances between patients indicate similar biomarker profiles, while larger distances indicate large differences between profiles. A) A heatmap demonstrated the pairwise Euclidian distance between the cohort’s complete plasma biomarker profiles. The profiles of the healthy controls are homogenous within themselves. The CKD/ESKD profiles are similar within themselves but different from the other cohorts. The AKI (COVID-19) cohort profiles are different from both cohorts and within themselves. B) A heatmap demonstrated the pairwise Euclidian distance between the cohort’s complete urine biomarker profiles. The profiles of healthy controls are similar within themselves. The inter-cohort comparison shows all patients having distinct profiles without any clear observable similarities.

**Supplementary Figure 1.**

**
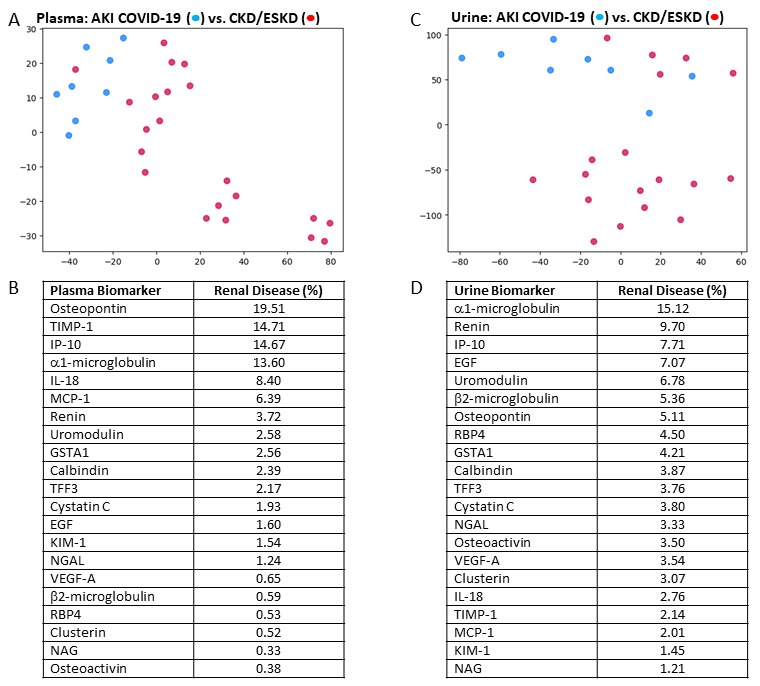
**

**Supplementary Figure 2.**

**
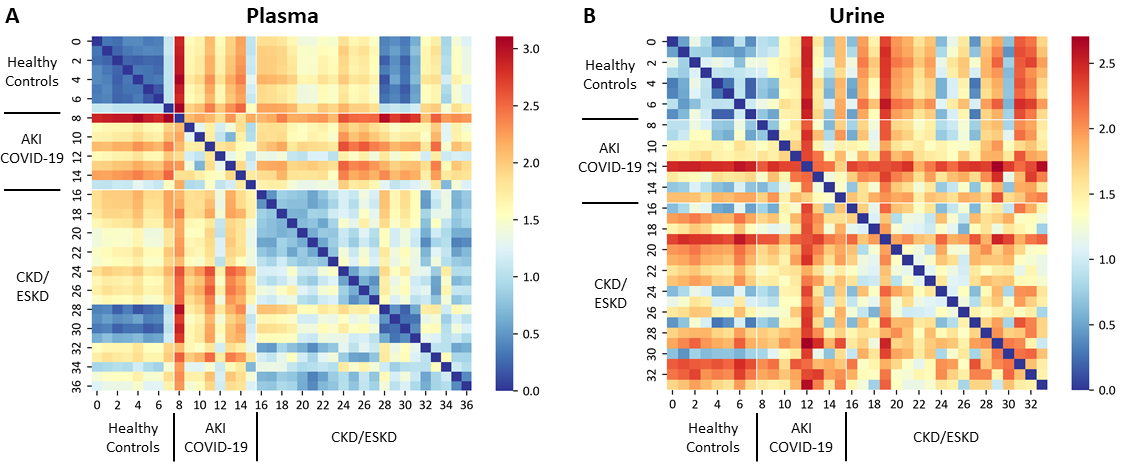
**
